# Supplementary material for: Aberrant splicing and defective mRNA production induced by somatic spliceosome mutations in myelodysplasia
Source: Nat Commun. 2018 Sep 7;9:3649. doi: 10.1038/s41467-018-06063-x (PMC6128865; doi:10.1038/s41467-018-06063-x)
Supplement: Supplementary file 1 — Supplementary Information [file 41467_2018_6063_MOESM1_ESM.pdf]

Supplementary Appendix

Aberrant splicing and defective mRNA production induced by somatic spliceosome mutations in myelodysplasia

Yusuke Shiozawa, Luca Malcovati, Anna Gallì, Aiko Sato-Otsubo, Keisuke Kataoka, Yusuke Sato, Yosaku Watatani, Hiromichi Suzuki, Tetsuichi Yoshizato, Kenichi Yoshida, Masashi Sanada, Hideki Makishima, Yuichi Shiraishi, Kenichi Chiba, Eva Hellström-Lindberg, Satoru Miyano, Seishi Ogawa, and Mario Cazzola.

Table of Contents

1. Supplementary Figures **5**

Supplementary Figure 1. Lndscape of genetic lesions in myeloid neoplasms with myelodysplasia 6

Supplementary Figure 2. Number of RNA sequencing reads 8

Supplementary Figure 3. Relative expression of mutant *SF3B1*-associated alternative 3′ splice sites.... 9

Supplementary Figure 4. Features of differentially spliced introns in *SF3B1*-mutated samples 11

Supplementary Figure 5. Sequence motifs of differentially spliced introns in *SF3B1*-mutated samples 12

Supplementary Figure 6. Proportion of sequencing reads mapped to intronic regions 13

Supplementary Figure 7. Fraction of tumor cells in cases with both *SF3B1* and *SRSF2* mutations. 14

Supplementary Figure 8. PSI values of mutant *SF3B1*- and *SRSF2*-associated alternative splicing events 15

Supplementary Figure 9. PSI values of mutant *U2AF1*-associated alternative splicing events 16

Supplementary Figure 10. Association between alternative splicing events and *TET2* co-mutation..... 17

Supplementary Figure 11. Distance of mutant *SF3B1*-associated alternative 3′ splice sites from canonical ones 18

Supplementary Figure 12. Differential gene expression analysis of *SF3B1*-mutated CD34+ cells 19

Supplementary Figure 13. Differential gene expression analysis of *SRSF2*-mutated CD34+ cells 21

Supplementary Figure 14. Increased expression of physiological targets of nonsense-mediated decay after cycloheximide treatment 23

Supplementary Figure 15. Nonsense-mediated decay of mutant *SF3B1*-associated aberrant transcripts 24

Supplementary Figure 16. Nonsense-mediated decay of mutant *SRSF2*-associated aberrant transcripts 26

Supplementary Figure 17. Confirmation of *SF3B1* mutations in CRISPR clones by DNA sequencing28

Supplementary Figure 18. Comparison of PSI values between primary samples and CRISPR cell lines 29

Supplementary Figure 19. Differential gene expression analysis comparing between the CRISPR cell lines with and without *SF3B1*^{K700E} mutation 31

Supplementary Figure 20. *In vitro* validation of mutant *SF3B1*-associated reduction of intron retention 32

Supplementary Figure 21. Electropherogram of cytoplasmic and nuclear RNA 33

Supplementary Figure 22. Relationship between gene expression levels and PSI values 34
Supplementary Figure 23. Usage of the *SF3B1*-associated abnormal 3′ splice sites in erythroid cells .36
Supplementary Figure 24. Mutant *SF3B1*-associated alternative 3′ splice sites in genes related to heme biosynthesis 37
Supplementary Figure 25. Functional significance of the altered 5′-untranslated region of *TMEM14C* 38
Supplementary Figure 26. In vitro validation of mutant *SF3B1*-induced abnormal splicing................39
Supplementary Figure 27. Mutant *SRSF2*-associated alternative exon usage in *EZH2* 41
Supplementary Figure 28. Cloning of the entire coding region of *EZH2* 42
Supplementary Figure 29. Up-regulation of the targets of polycomb repressive complex 2 in *SRSF2*-mutated CD34+ cells 43
Supplementary Figure 30. Examples of mutant *SRSF2*-associated alternative exon usage 44
Supplementary Figure 31. Usage of the *EZH2* cryptic exon in HeLa cells transduced with *U2AF1* S34F mutant 45
Supplementary Figure 32. Mutant *U2AF1*-associated alterations in 3′ splice site consensus sequences 46
Supplementary Figure 33. Uncropped gel images of RT-PCR gels of differentially spliced introns 48
Supplementary Figure 34. Uncropped gel images of RT-PCR gels of the differentially spliced sites of *ABCB7* and *PPOX* 49
Supplementary Figure 35. Uncropped images of immunoblots of CRISPR cell lines..........................50

2. Supplementary Tables **51**
Supplementary Table 1. Patient characteristics 51
Supplementary Table 2. Gene list for targeted deep sequencing..53
Supplementary Table 3. Number of SF-mutated patients with or without mutations in epigenetic regulators 54
Supplementary Table 4. Off-targets and primer sequences 55
Supplementary Table 5. Primer sequences for RT-PCR, PCR cloning, qRT-PCR, and 5′-RACE........56
Supplementary Table 6. Immunoblots primary antibodies, dilutions used in experiments, company, and catalogue information. 57

3. Supplementary Methods **58**
Variant filtering criteria 58

Target genes of the polycomb repressive complex 2 58

4. Supplementary References 61

1. Supplementary Figures

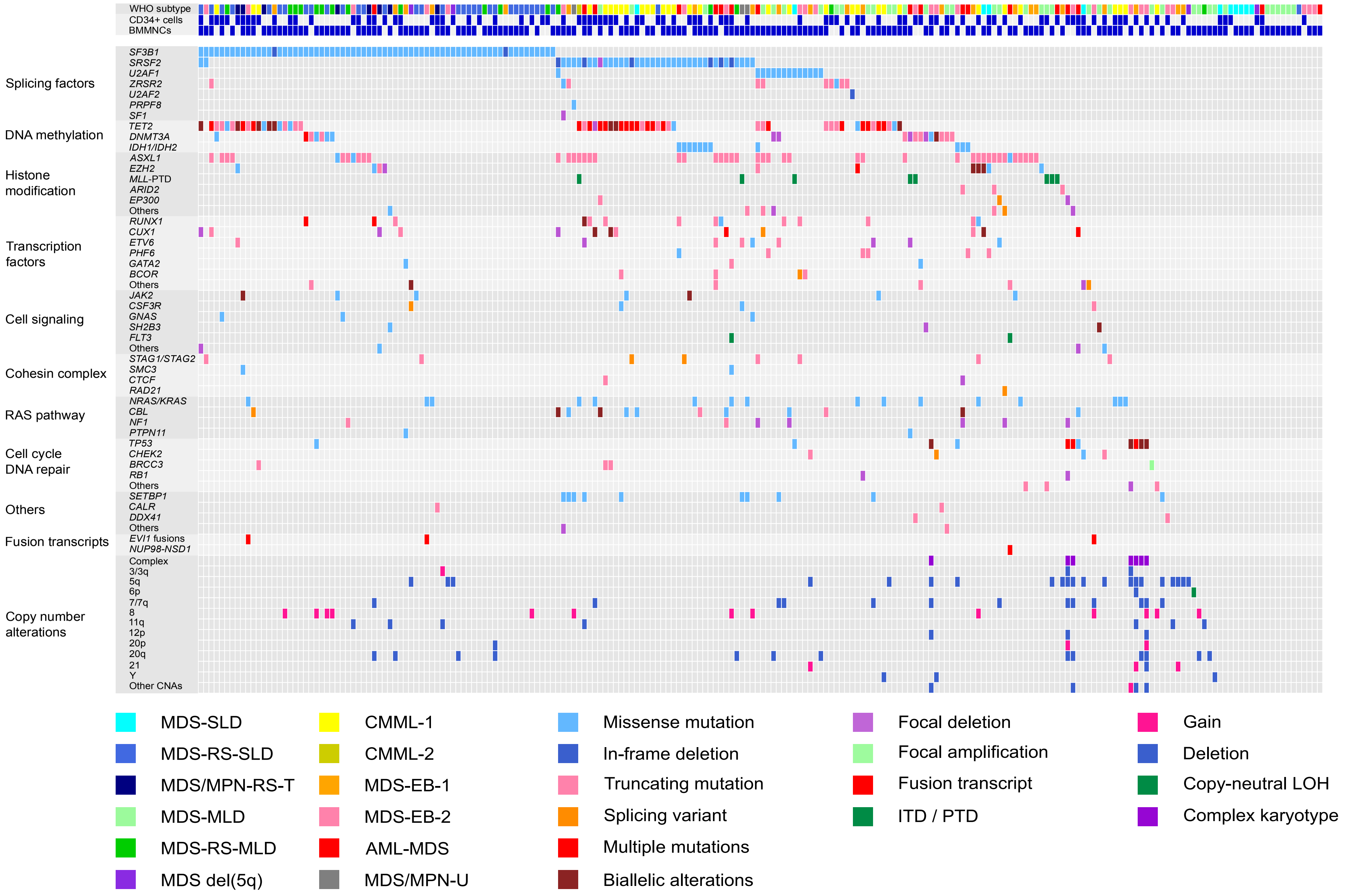
WHO subtype
CD34+ cells
BMMNCs
Splicing factors
SF3B1
SRSF2
U2AF1
ZRSR2
U2AF2
PRPF8
SF1
DNA methylation
TET2
DNMT3A
IDH1/IDH2
Histone modification
ASXL1
EZH2
MLL-PTD
ARID2
EP300
Others
Transcription factors
RUNX1
CUX1
ETV6
PHF6
GATA2
BCOR
Others
Cell signaling
JAK2
CSF3R
GNAS
SH2B3
FLT3
Others
Cohesin complex
STAG1/STAG2
SMC3
CTCF
RAD21
RAS pathway
NRAS/KRAS
CBL
NF1
PTPN11
Cell cycle DNA repair
TP53
CHEK2
BRCC3
RB1
Others
Others
SETBP1
CALR
DDX41
Others
Fusion transcripts
EVI1 fusions
NUP98-NSD1
Copy number alterations
Complex
3/3q
5q
6p
7/7q
8
11q
12p
20p
20q
21
Y
Other CNAs
MDS-SLD
MDS-RS-SLD
MDS/MPN-RS-T
MDS-MLD
MDS-RS-MLD
MDS del(5q)
CMML-1
CMML-2
MDS-EB-1
MDS-EB-2
AML-MDS
MDS/MPN-U
Missense mutation
In-frame deletion
Truncating mutation
Splicing variant
Multiple mutations
Biallelic alterations
Focal deletion
Focal amplification
Fusion transcript
ITD / PTD
Gain
Deletion
Copy-neutral LOH
Complex karyotype

Supplementary Figure 1. Landscape of genetic lesions in myeloid neoplasms with myelodysplasia

WHO subtypes, sources of RNA, and types of genetic lesions identified by targeted deep sequencing and RNA sequencing. Splicing variants indicate variants that disrupt splicing. Biallelic alterations are inactivating mutations concomitant with uniparental disomy, those with heterozygous deletions in the same clone, or biallelic deletions. MDS-SLD, MDS with single lineage dysplasia; MDS-RS-SLD, MDS with ring sideroblasts with single lineage dysplasia; MDS/MPN-RS-T, MDS/MPN with ring sideroblasts and thrombocytosis; MDS-MLD, MDS with multilineage dysplasia; MDS-RS-MLD, MDS with ring sideroblasts with multilineage dysplasia; CMML, chronic myelomonocytic leukemia; MDS-EB, MDS with excess blasts; AML-MDS, AML with myelodysplasia-related changes; MDS/MPN-U, MDS/MPN, unclassifiable.; ITD, internal tandem duplication; PTD, partial tandem duplication; LOH, loss of heterozygosity.

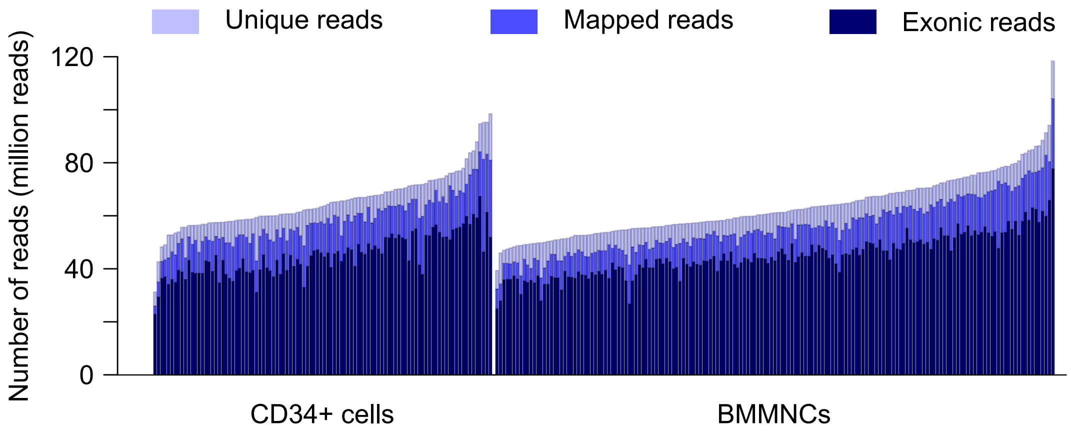

Supplementary Figure 2. Number of RNA sequencing reads

A barplot shows number of sequence reads after removal of PCR duplicates (unique reads), reads mapped to the human reference genome (hg19), and reads mapped to exonic regions. Each bar represents one sample. Bone marrow CD34+ cells and BMMNCs are separately shown.

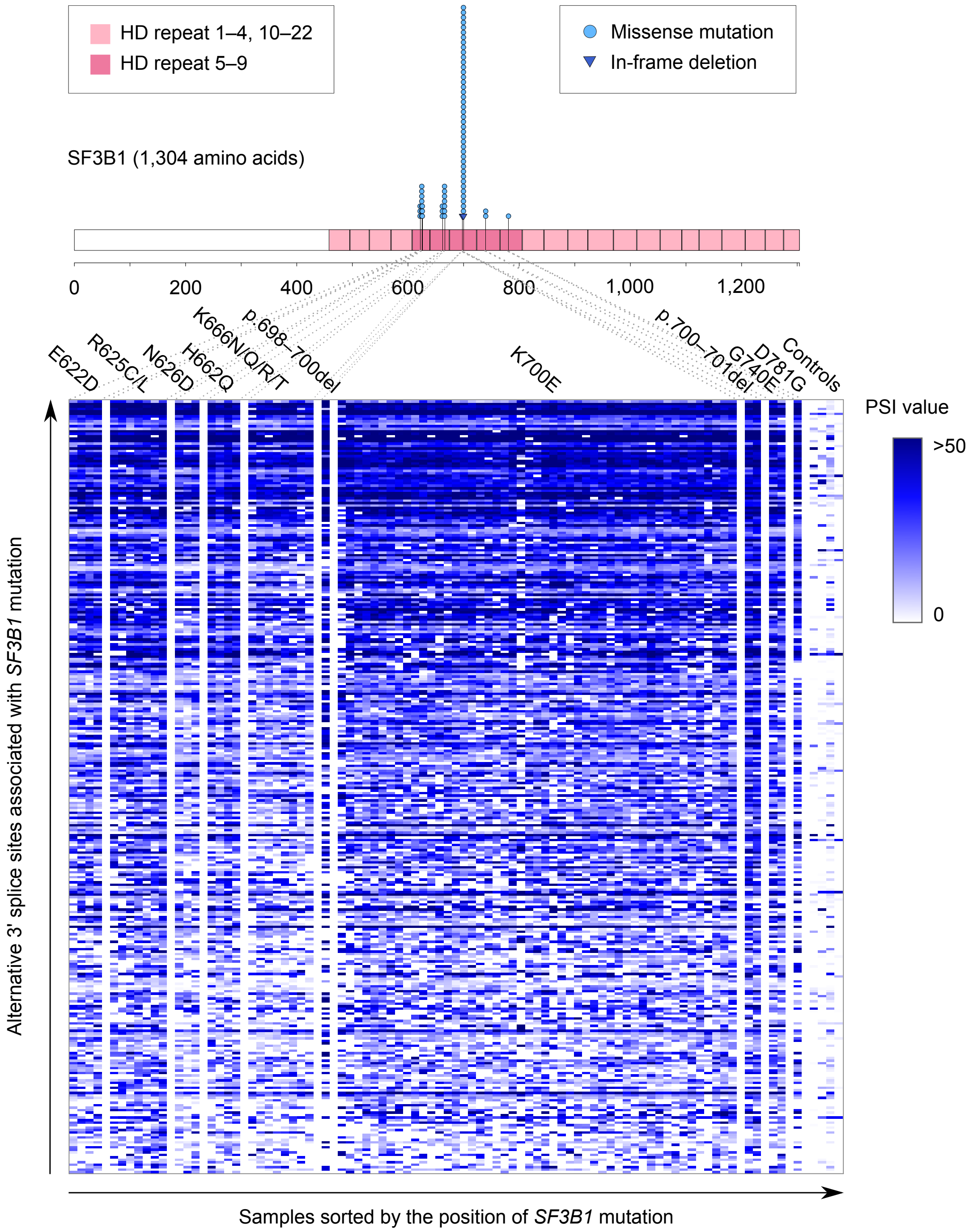

Supplementary Figure 3. Relative expression of mutant *SF3B1*-associated alternative 3′ splice sites

The upper panel shows positions of *SF3B1* mutations. Mutations were clustered at several amino acids in 5–9th heat domains (HDs). A circle and a triangle indicate missense mutation and in-frame deletion, respectively. Two in-frame deletions involving K700 were found in our cohort. The lower heatmap shows PSI values of alternative 3′ splice sites that were associated with *SF3B1* mutation both in bone marrow

CD34 + cells and BMMNCs. Each row represents one alternative splicing event and each column represents one sample. Samples are sorted according to the position of *SF3B1* mutation. The left four columns indicate mean PSI values in four control groups: 1) bone marrow CD34+ cell samples of myelodysplasia patients without splicing factor mutations, 2) BMMNC samples of myelodysplasia patients without splicing factor mutations, 3) bone marrow CD34+ cell samples obtained from healthy adults, and 4) BMMNC samples obtained from healthy adults.

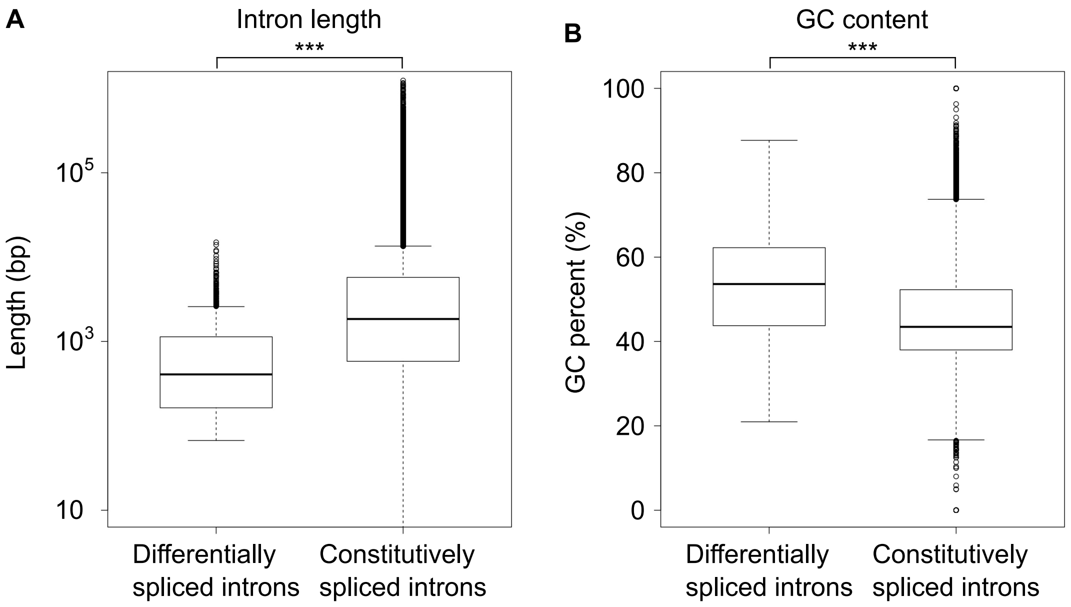

Supplementary Figure 4. Features of differentially spliced introns in *SF3B1*-mutated samples

Boxplots show distribution of intron length (A) and GC content (B) in differentially spliced introns in *SF3B1*-mutated samples and constitutively spliced introns. ***: *P*<0.001.

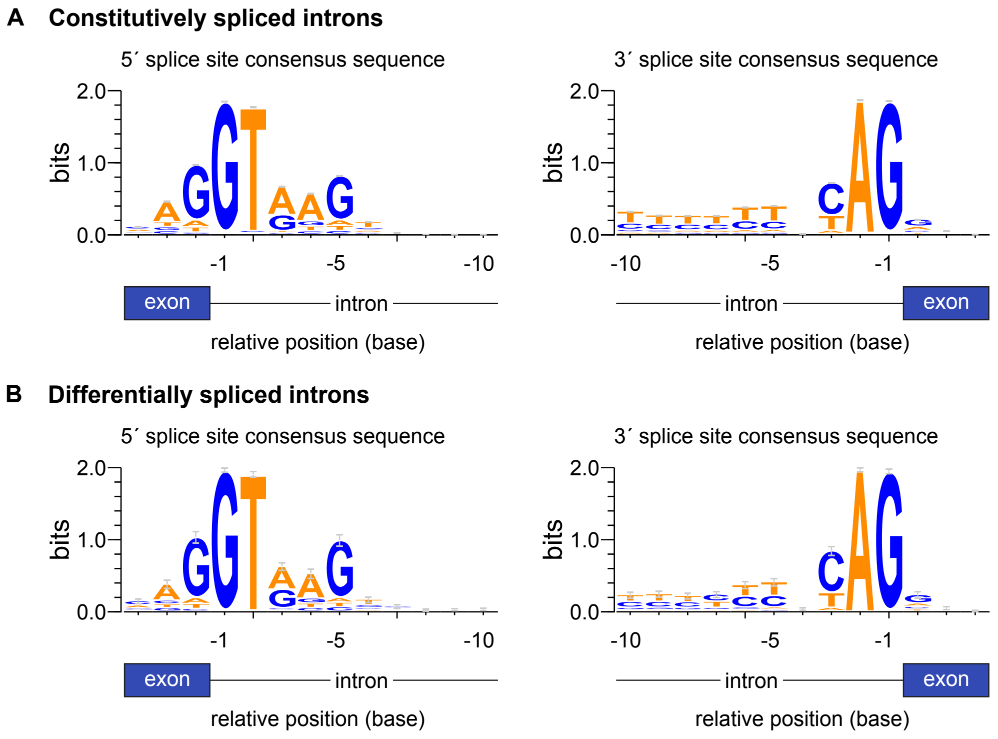

Supplementary Figure 5. Sequence motifs of differentially spliced introns in *SF3B1*-mutated samples

Consensus sequences around 5´ and 3´ splice sites of constitutively spliced introns (A) and differentially spliced introns (B). Horizontal axis denotes genomic coordinates defined with respect to the 5´ and 3´ splice sites. Vertical axis indicates information content in bits.

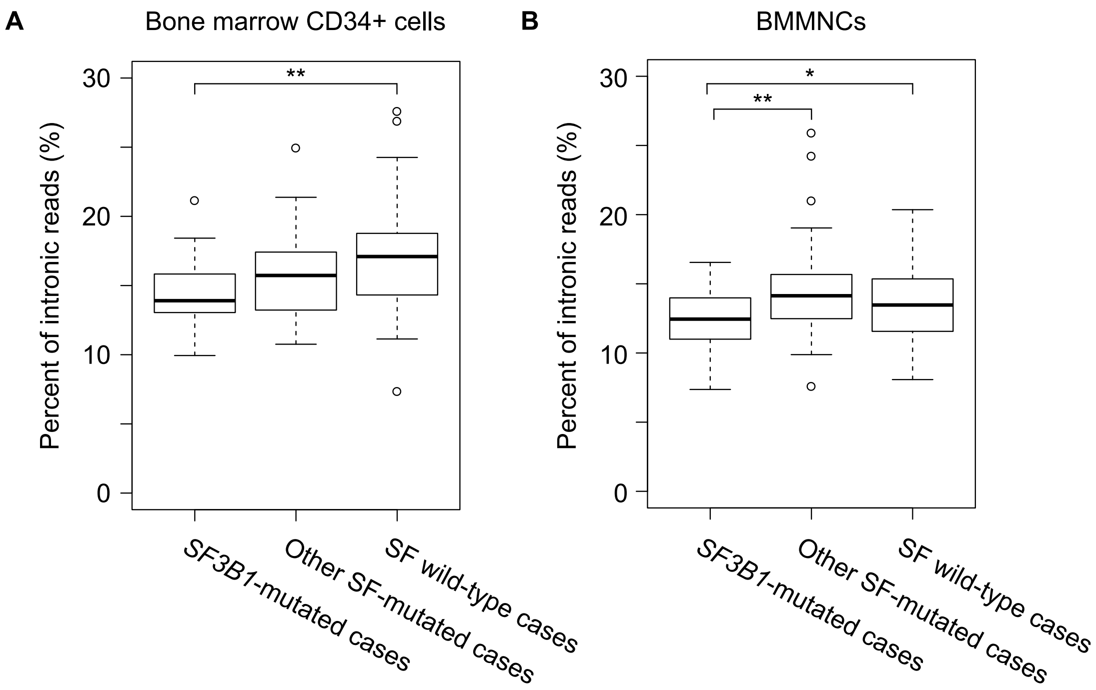

Supplementary Figure 6. Proportion of sequencing reads mapped to intronic regions

Boxplots show percent of sequencing reads mapped to intronic regions in bone marrow CD34+ cell samples (A) and BMMNCs (B). Boxes are drawn for *SF3B1*-mutated patients, those with other splicing factor (SF) mutations, and those without SF mutations. *: adjusted $P<0.05$; **: adjusted $P<0.01$.

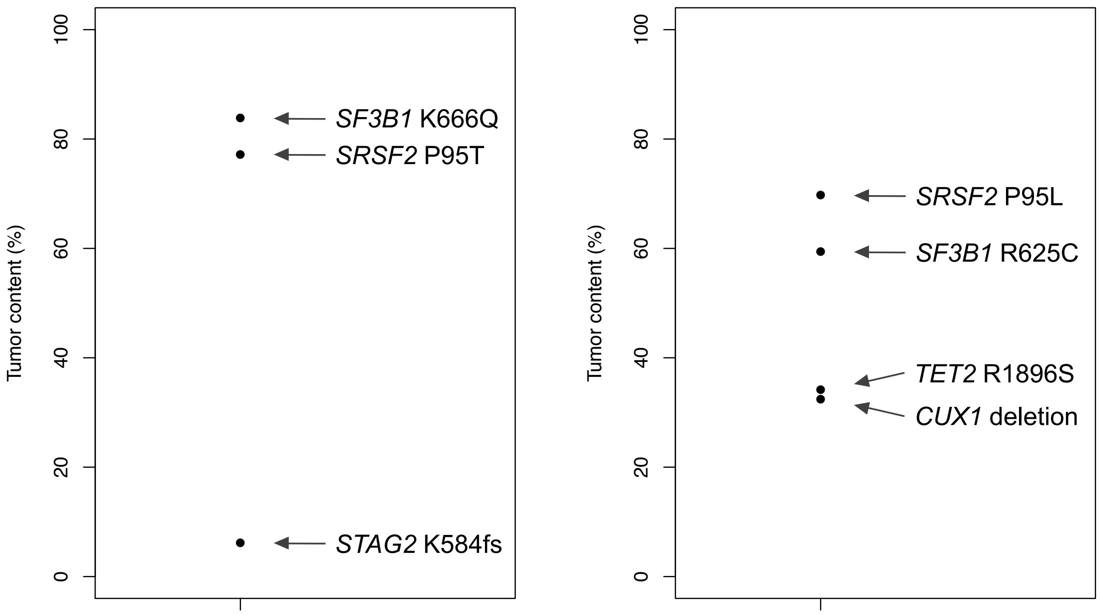

Supplementary Figure 7. Fraction of tumor cells in cases with both *SF3B1* and *SRSF2* mutations.

Dotplots show fraction of tumor cells with various mutations in two patients with both *SF3B1* and *SRSF2* mutations. Tumor fraction was estimated from variant allele frequencies adjusted for genomic copy numbers. Because *SF3B1* and *SRSF2* mutations are present in >50% of tumor cells in either case, at least some clones must contain both mutations.

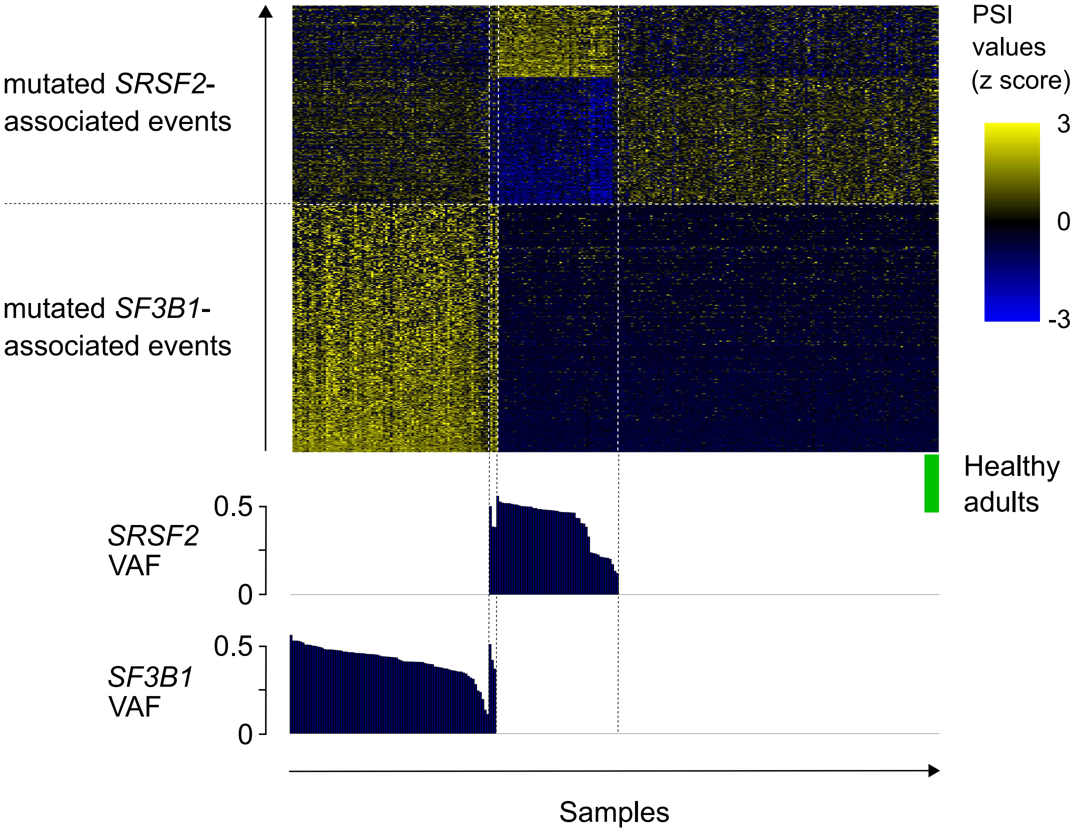

Supplementary Figure 8. PSI values of mutant *SF3B1*- and *SRSF2*-associated alternative splicing events

A heatmap shows PSI values of mutant *SF3B1*- and *SRSF2*-associated alternative splicing events. Columns and rows correspond to samples and splice alterations, respectively. Variant allele frequencies (VAFs) of *SF3B1* and *SRSF2* mutations are shown below the heatmap. Three samples from two patients had both *SF3B1* and *SRSF2* mutations.

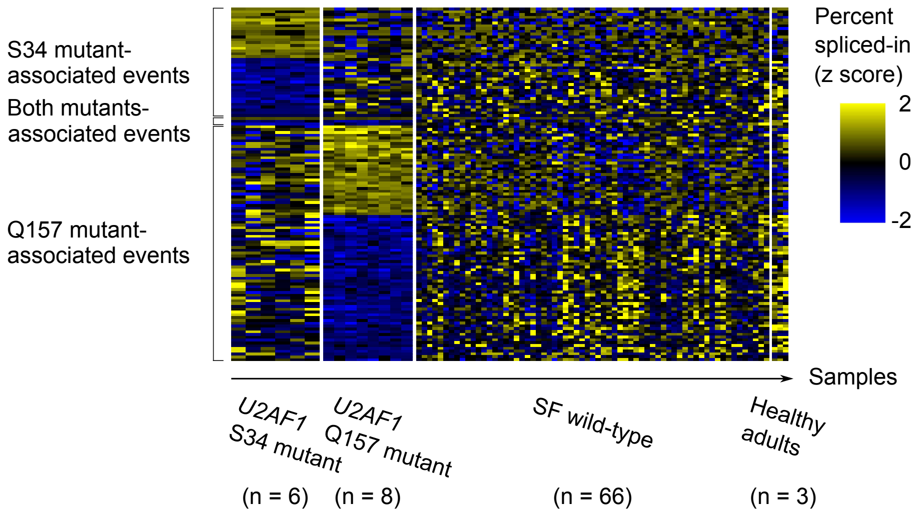

Supplementary Figure 9. PSI values of mutant *U2AF1*-associated alternative splicing events

A heatmap shows PSI values of mutant *U2AF1*-associated alternative splicing events: those associated with *U2AF1* S34 mutation (n = 39), those with *U2AF1* Q157 mutation (n = 84), and those with both mutations (n = 3). Columns and rows correspond to samples and splice alterations, respectively. Samples are sorted according to the mutation status of *U2AF1*.

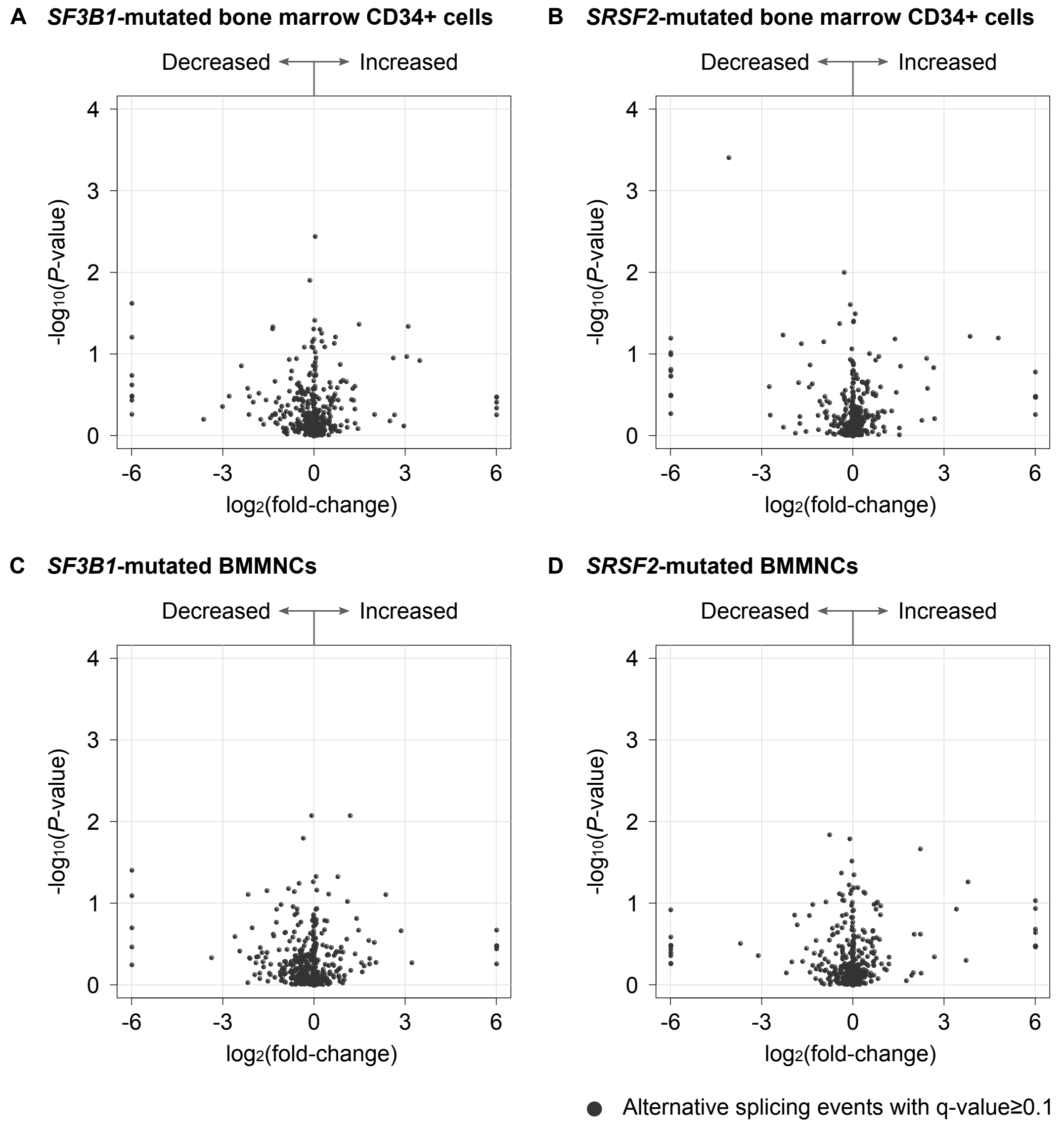

Supplementary Figure 10. Association between alternative splicing events and *TET2* co-mutation

Volcano plots comparing PSI values between each SF-mutated samples with and without co-mutations in *TET2*: *SF3B1*-mutated bone marrow CD34+ cells (A), *SRSF2*-mutated bone marrow CD34+ cells (B), *SF3B1*-mutated BMMNCs (C), and *SRSF2*-mutated BMMNCs (D). Alternative splicing events were separately plotted for *SF3B1*- or *SRSF2*-mutated bone marrow CD34+ cells and BMMNCs. X-axis indicates fold changes in PSI values on a $\log_2$ scale. Y-axis indicates *P* values on a negative $\log_{10}$ scale. No event reached statistical significance of q-value <0.1.

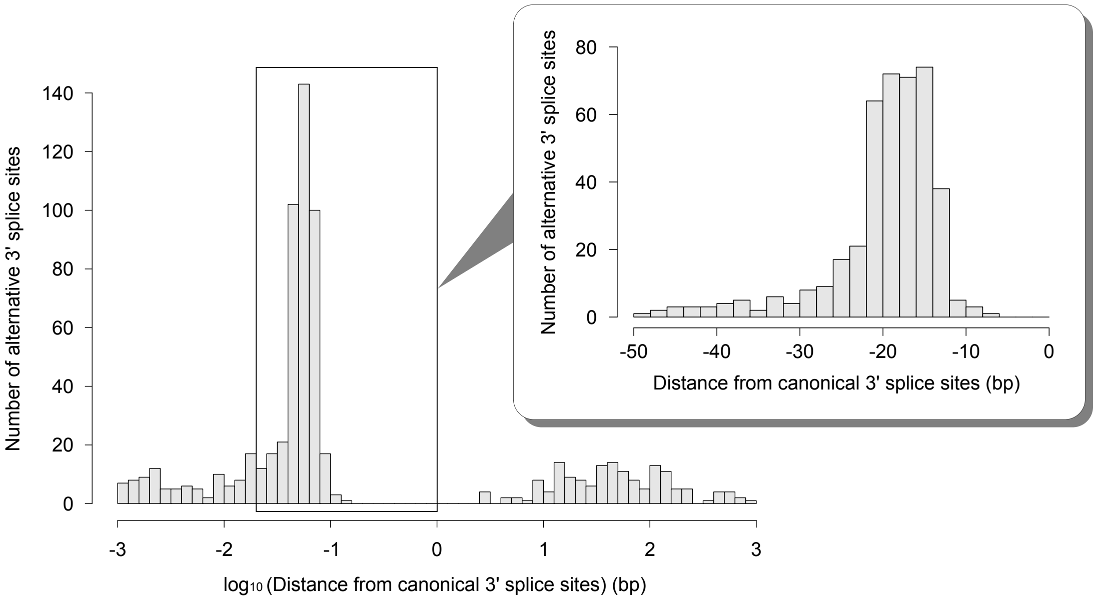

Supplementary Figure 11. Distance of mutant *SF3B1*-associated alternative 3´ splice sites from canonical ones

A histogram shows distribution of alternative 3´ splice sites relative to their corresponding canonical 3´ splice sites. X-axis indicates a distance on a $\log_{10}$ scale. Positive and negative values denote a shift upstream and downstream from canonical sites, respectively. Enlarged view shows the number of alternative 3´ splice sites located at 0–50 bp upstream of the canonical 3´ splice sites.

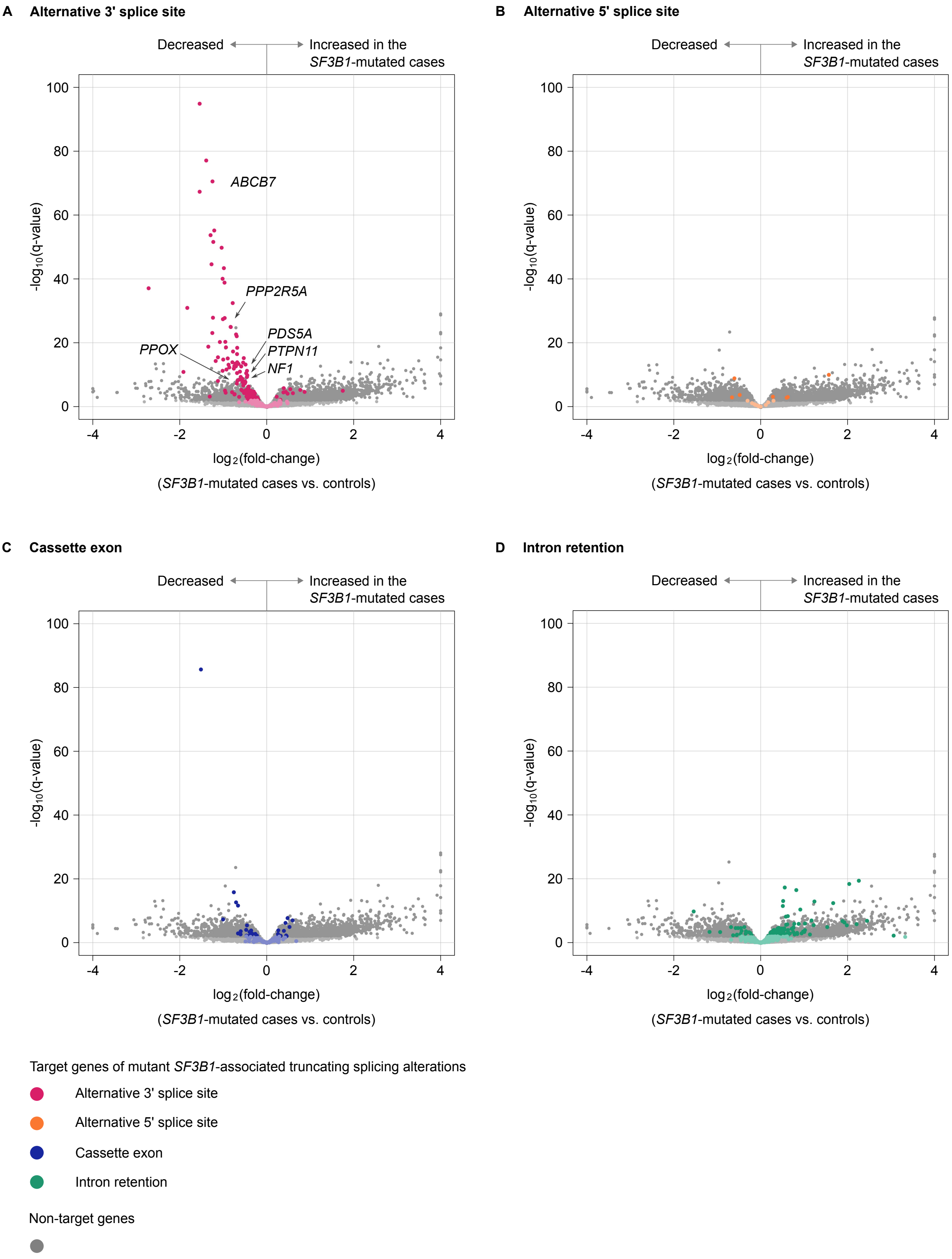
A Alternative 3' splice site
B Alternative 5' splice site
C Cassette exon
D Intron retention
Decreased
Increased in the SF3B1-mutated cases
-log10(q-value)
100
80
60
40
20
0
-4
-2
0
2
4
log2(fold-change)
(SF3B1-mutated cases vs. controls)
ABCB7
PPP2R5A
PDS5A
PTPN11
NF1
PPOX
Target genes of mutant SF3B1-associated truncating splicing alterations
Alternative 3' splice site
Alternative 5' splice site
Cassette exon
Intron retention
Non-target genes

Supplementary Figure 12. Differential gene expression analysis of *SF3B1*-mutated CD34+ cells

Volcano plots compare gene expression levels between the *SF3B1*-mutated CD34+ cells and those without known splicing factor mutation. X-axis indicates fold changes in gene expression on a $\log_2$ scale. Y-axis indicates q-values on a negative $\log_{10}$ scale. The expression level of transcripts without truncating splicing alterations was estimated for the target genes of *SF3B1* mutation-associated splicing alterations. The plots are depicted for the non-target genes and the target ones of alternative 3´ splice sites (A), alternative 5´ splice sites (B), cassette exon (C), and intron retention (D).

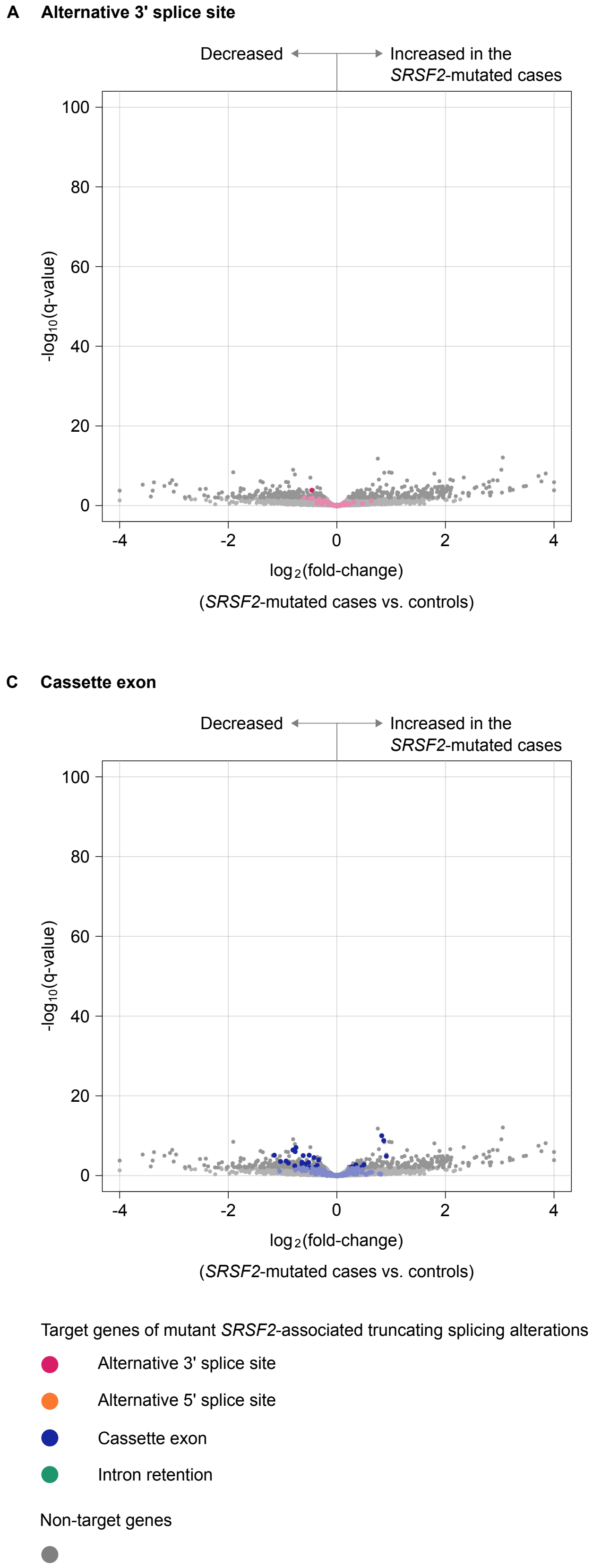

A Alternative 3' splice site
B Alternative 5' splice site
C Cassette exon
D Intron retention
Decreased
Increased in the SRSF2-mutated cases
-log10(q-value)
100
80
60
40
20
0
-4
-2
0
2
4
log2(fold-change)
(SRSF2-mutated cases vs. controls)
Target genes of mutant SRSF2-associated truncating splicing alterations
Alternative 3' splice site
Alternative 5' splice site
Cassette exon
Intron retention
Non-target genes

Supplementary Figure 13. Differential gene expression analysis of *SRSF2*-mutated CD34+ cells

Volcano plots compare gene expression levels between the *SRSF2*-mutated CD34+ cells and those without known splicing factor mutation. X-axis indicates fold changes in gene expression on a $\log_2$ scale. Y-axis indicates q-values on a negative $\log_{10}$ scale. The expression level of transcripts without truncating splicing alterations was estimated for the target genes of *SRSF2* mutation-associated splicing alterations. The plots are depicted for the non-target genes and the target ones of alternative 3´ splice sites (A), alternative 5´ splice sites (B), cassette exon (C), and intron retention (D).

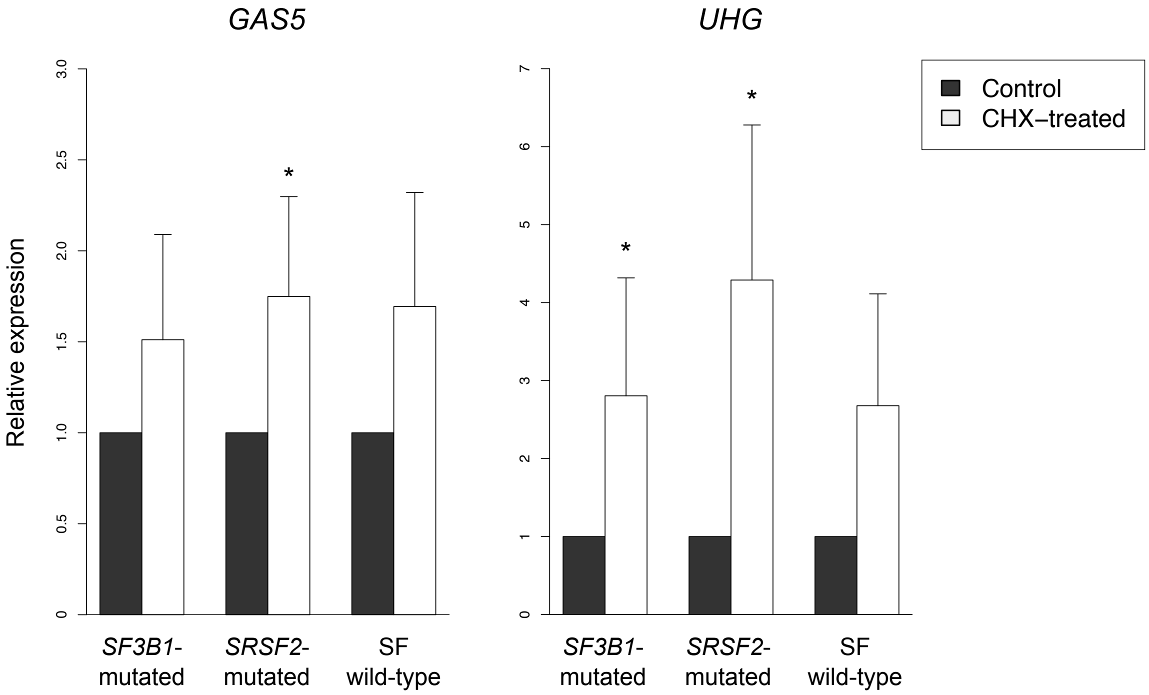

Supplementary Figure 14. Increased expression of physiological targets of nonsense-mediated decay after cycloheximide treatment

Barplots show the levels of *GAS5*, and *UHG* in bone marrow CD34+ cells from MDS patients, each with and without cycloheximide (CHX) treatment. Error bars are standard deviation. *: $P<0.05$ by paired t-test.

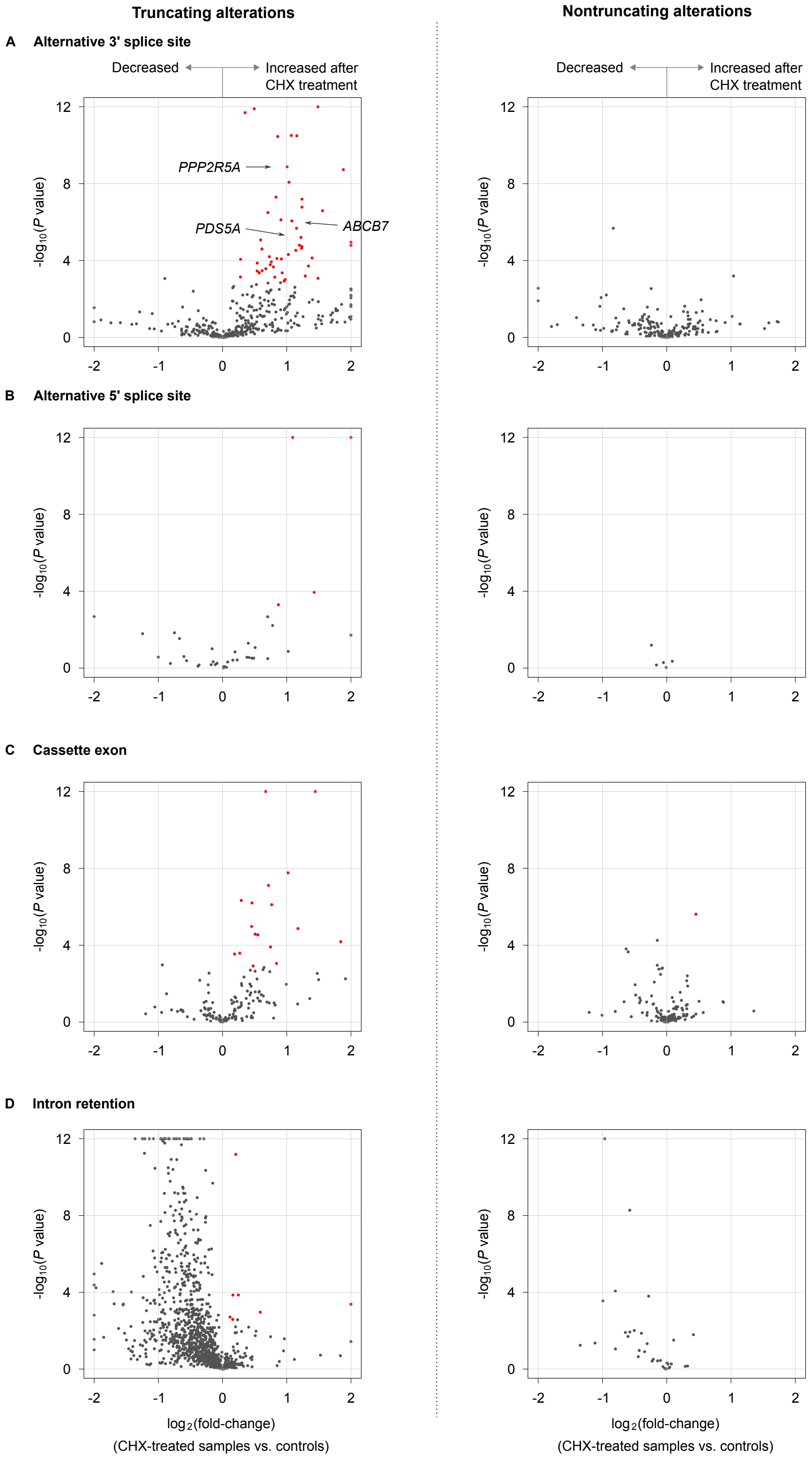
Truncating alterations
Nontruncating alterations
A Alternative 3' splice site
Decreased
Increased after CHX treatment
PPP2R5A
PDS5A
ABCB7
-log10(P value)
B Alternative 5' splice site
C Cassette exon
D Intron retention
log2(fold-change)
(CHX-treated samples vs. controls)

Supplementary Figure 15. Nonsense-mediated decay of mutant *SF3B1*-associated aberrant transcripts

Volcano plots compare PSI values of *SF3B1*-associated splicing alterations between samples with and without cycloheximide (CHX) treatment. The plots are separately depicted for alternative 3′ splice sites (A), alternative 5′ splice sites (B), cassette exon (C), and intron retention (D). The left and right plots show truncating and nontruncating alterations, respectively. X-axis indicates fold changes in read fractions after CHX treatment on a $\log_2$ scale. Y-axis indicates *P* values on a negative $\log_{10}$ scale. Transcripts increased after CHX treatment with q-value<0.01 are depicted in red. Transcripts exceeding the upper limit of Y-axis are plotted at the upper limit.

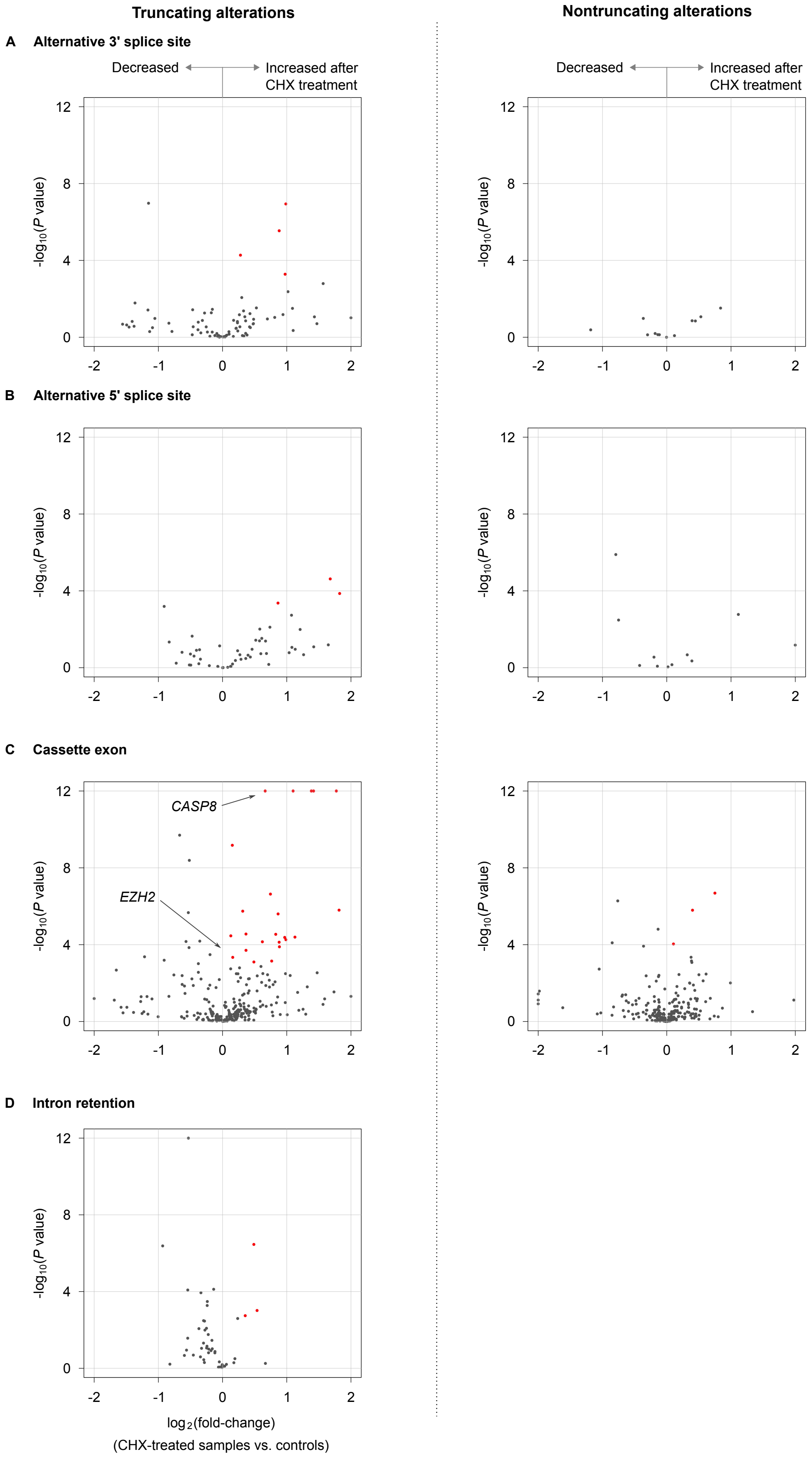
Truncating alterations
Nontruncating alterations
A
Alternative 3' splice site
Decreased
Increased after
CHX treatment
-log10(P value)
12
8
4
0
-2
-1
0
1
2
B
Alternative 5' splice site
C
Cassette exon
CASP8
EZH2
D
Intron retention
log2(fold-change)
(CHX-treated samples vs. controls)

Supplementary Figure 16. Nonsense-mediated decay of mutant *SRSF2*-associated aberrant transcripts

Volcano plots compare PSI values of *SRSF2*-associated splicing alterations between samples with and without cycloheximide (CHX) treatment. The plots are separately depicted for alternative 3′ splice sites (A), alternative 5′ splice sites (B), cassette exon (C), and intron retention (D). The left and right plots show truncating and nontruncating alterations, respectively. X-axis indicates fold changes in read fractions after CHX treatment on a $\log_2$ scale. Y-axis indicates *P* values on a negative $\log_{10}$ scale. Transcripts increased after CHX treatment with q-value<0.01 are depicted in red. Transcripts exceeding the upper limit of Y-axis are plotted at the upper limit.

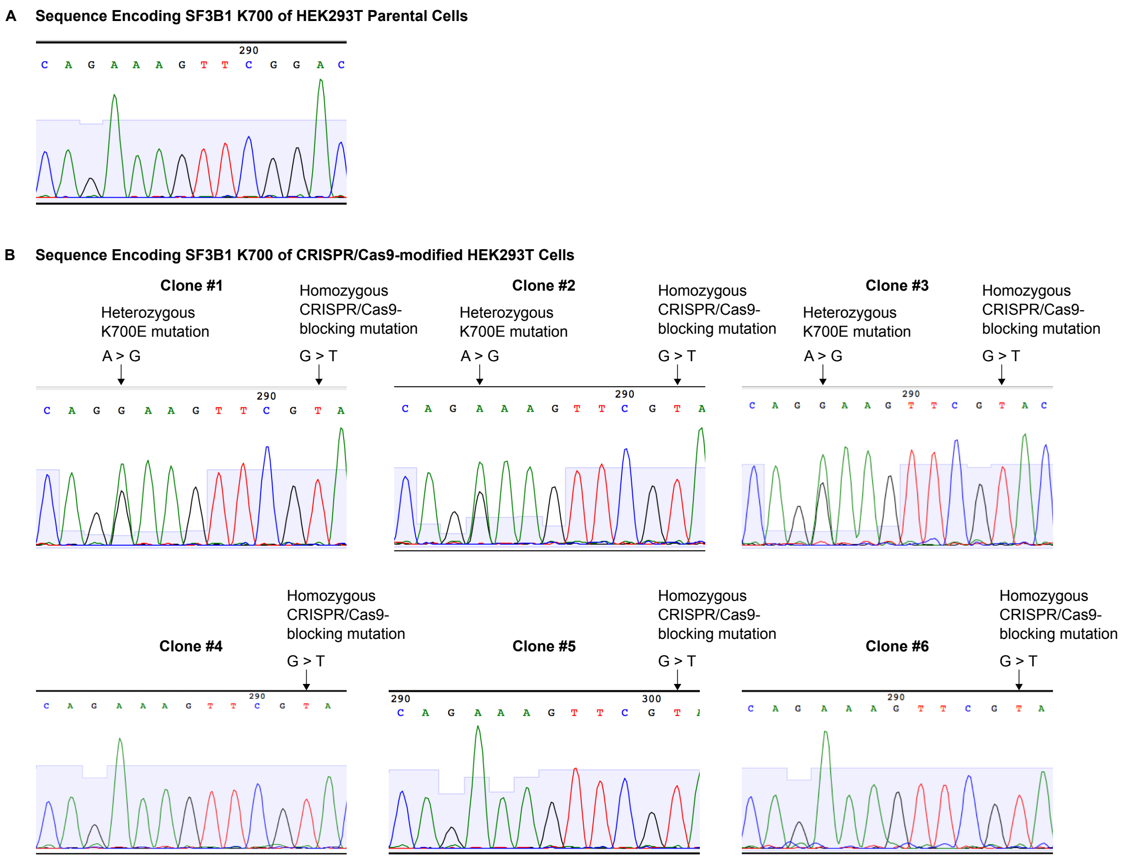

Supplementary Figure 17. Confirmation of *SF3B1* mutations in CRISPR clones by DNA sequencing

Sequencing chromatograms of the sequence encoding SF3B1 K700 of HEK293T parental cells (Panel A) and CRISPR/Cas9-modified ones (Panel B). The K700E mutation and synonymous CRISPR/Cas9-blocking mutation are indicated.

A Alternative 3´ splice sites

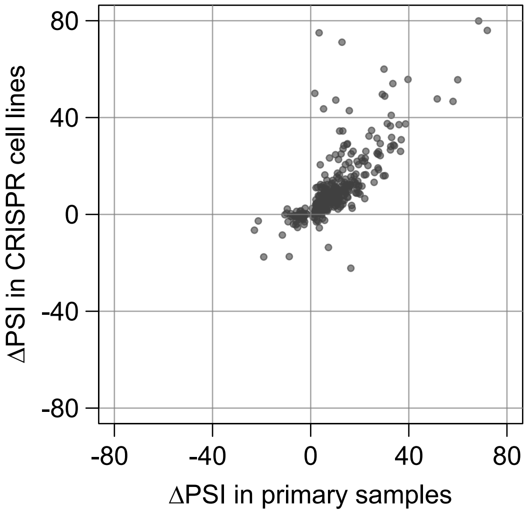

B Alternative 5´ splice sites

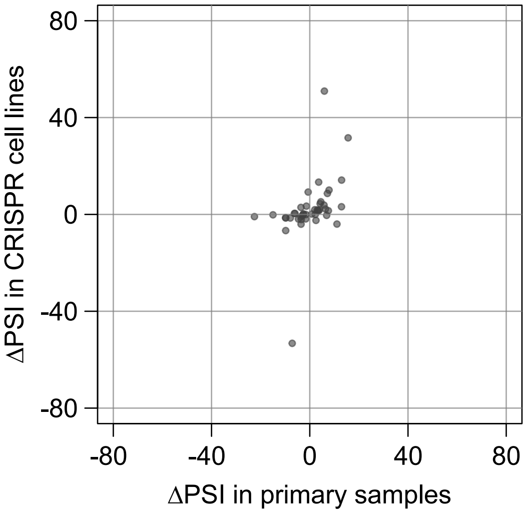

C Intron retention

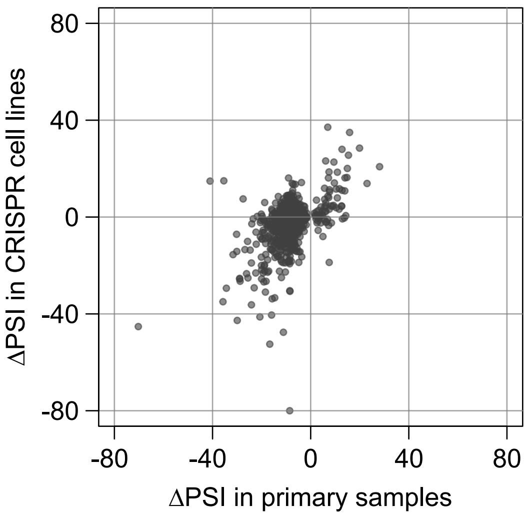

D Cassette exon

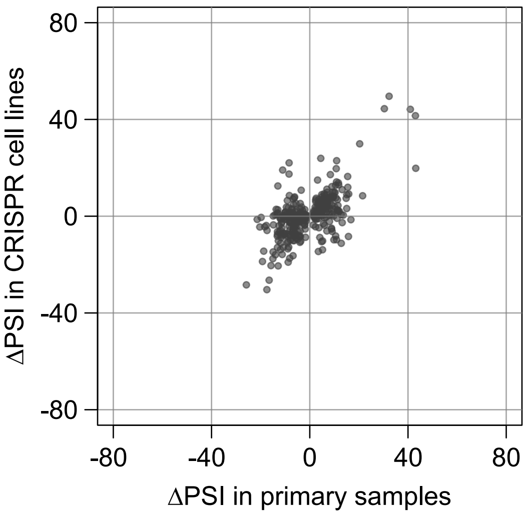

E Other types of alternative exon usage

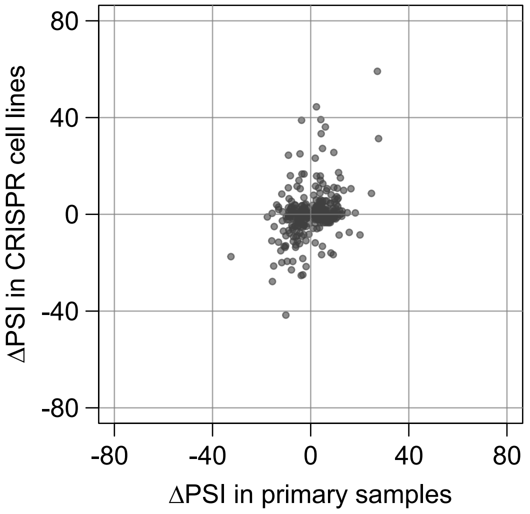

Supplementary Figure 18. Comparison of PSI values between primary samples and CRISPR cell lines

Scatter plots show differences in mean PSI values between *SF3B1*-mutated samples and those without SF mutations (ΔPSI values). Plots are depicted for mutant *SF3B1*-associated alternative splicing events. The plots are separately depicted for alternative 3′ splice sites (A), alternative 5′ splice sites (B), intron retention (C), cassette exon (D), and other types of alternative exon usage (E). ΔPSI values are compared between primary samples (X-axis) and CRISPR cell lines (Y-axis).

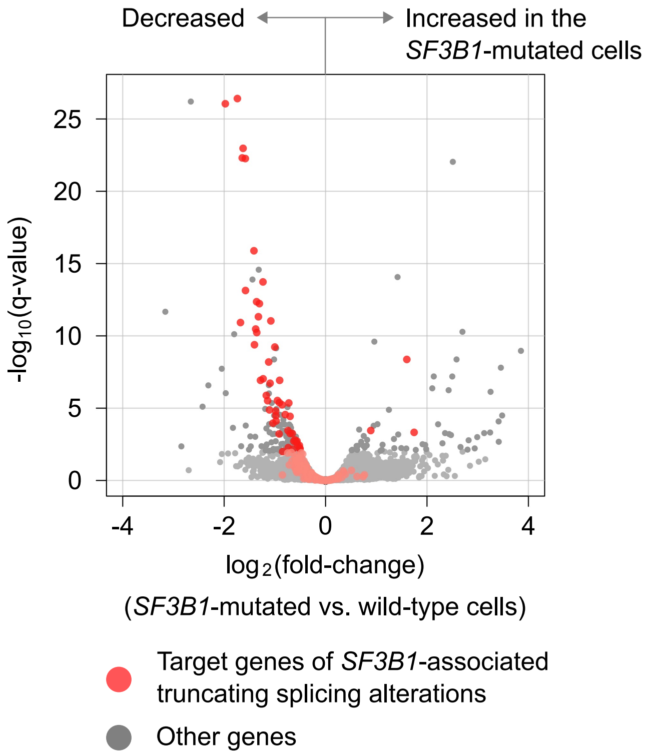

Supplementary Figure 19. Differential gene expression analysis comparing between the CRISPR cell lines with and without *SF3B1*$^{\text{K700E}}$ mutation

A volcano plot compares gene expression levels between the CRISPR cell lines with and without *SF3B1*$^{\text{K700E}}$ mutation. X-axis indicates fold changes in gene expression on a $\log_2$ scale. Y-axis indicates q-values on a negative $\log_{10}$ scale. The expression level of transcripts without truncating splicing alterations was estimated for the target genes of *SF3B1* mutation-associated alternative 3´ splice sites. The plots are depicted in red for the target genes of *SF3B1* mutation-associated alternative 3´ splice sites.

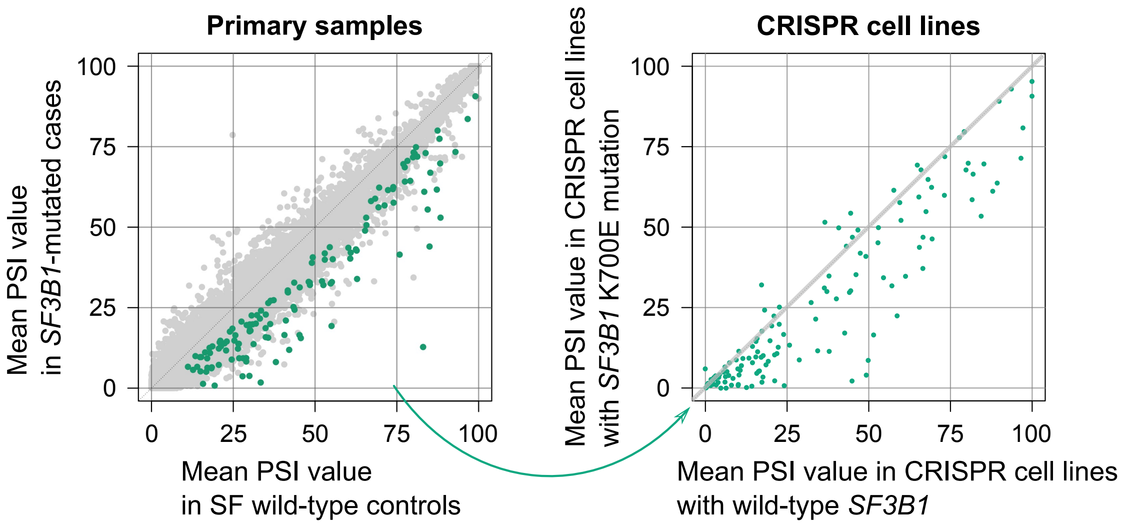

Supplementary Figure 20. ***In vitro*** **validation of mutant** ***SF3B1*****-associated reduction of intron retention**

Scatter plots showing mean PSI values of 134 intron retention events with highly significant reduction in *SF3B1*-mutated primary samples (q-value $<1\text{x}10^{-5}$). The left and right panels show mean PSI values in primary bone marrow samples and in CRISPR cell lines, respectively.

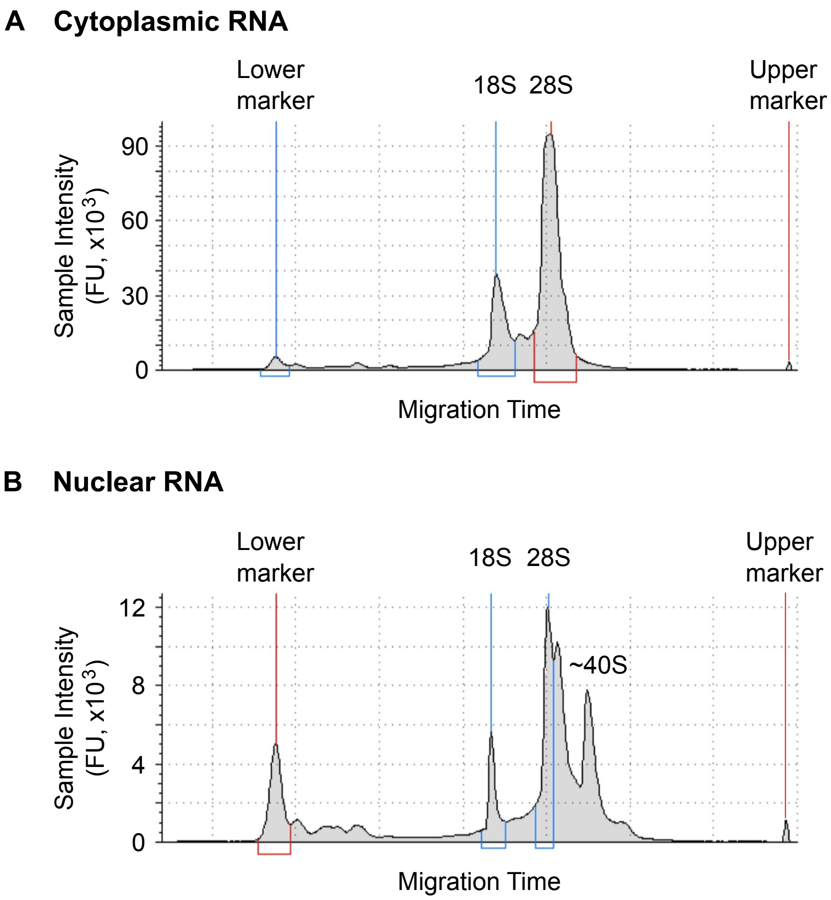

Supplementary Figure 21. Electropherogram of cytoplasmic and nuclear RNA

TapeStation® electropherogram of cytoplasmic (A) and nuclear (B) RNA. Horizontal and vertical axes indicate migration time and sample intensity, respectively.

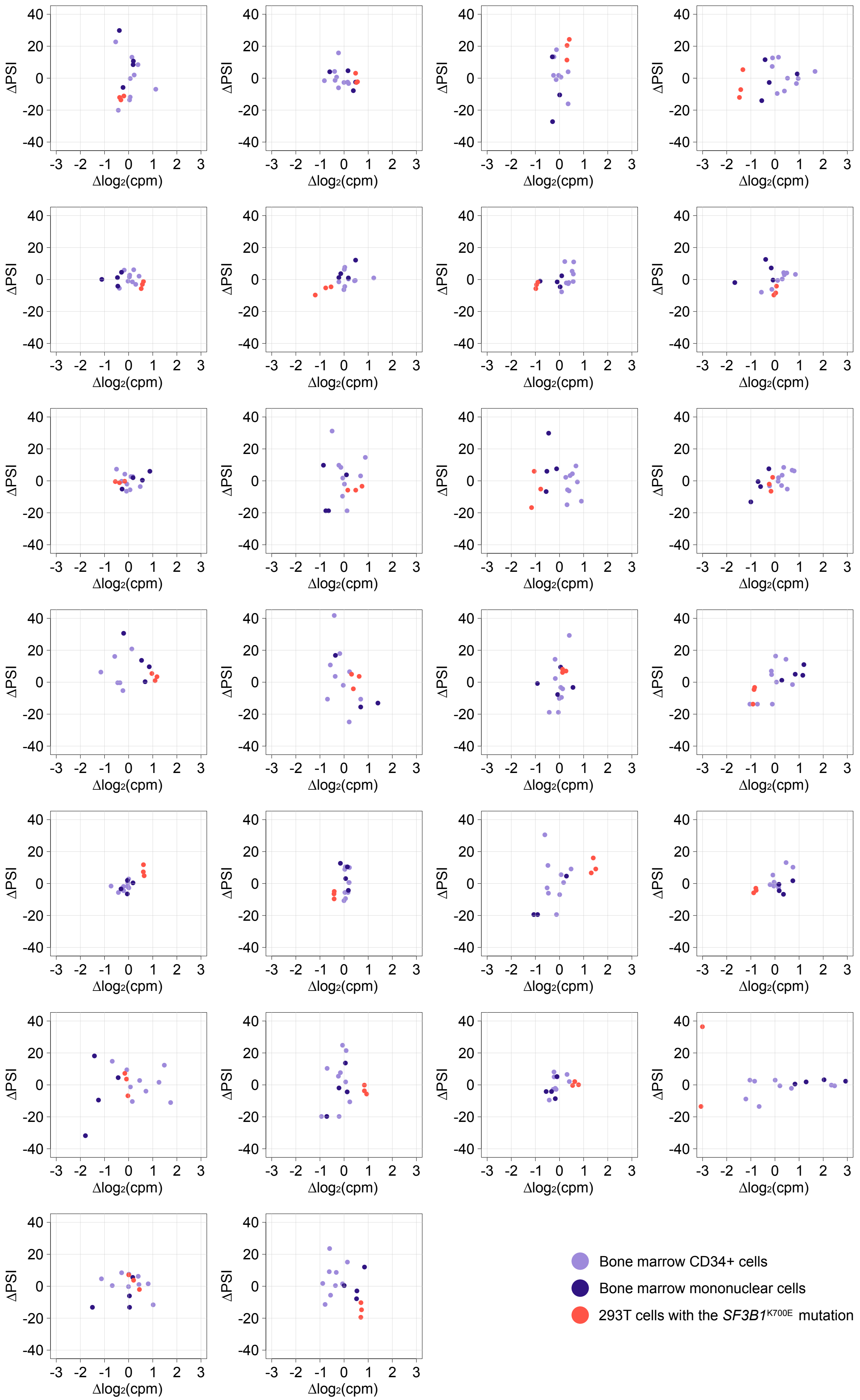
ΔPSI
Δlog2(cpm)
40
20
0
-20
-40
-3
-2
-1
0
1
2
3
Bone marrow CD34+ cells
Bone marrow mononuclear cells
293T cells with the *SF3B1*K700E mutation

Supplementary Figure 22. Relationship between gene expression levels and PSI values

Scatter plots comparing between gene expression levels and PSI values. Plots are depicted for 26 genes with mutant *SF3B1*-associated aberrant 3′ splice sites that fulfilled the criteria mentioned in the main text. X-axis indicates fold changes in gene expression levels on a $\log_2$ scale with a mean set to 0. Y-axis denotes PSI values with a mean set to 0. Primary bone marrow CD34+ cells, BMMNCs, and HEK293T cells are depicted in light blue, dark blue, and red circles, respectively.

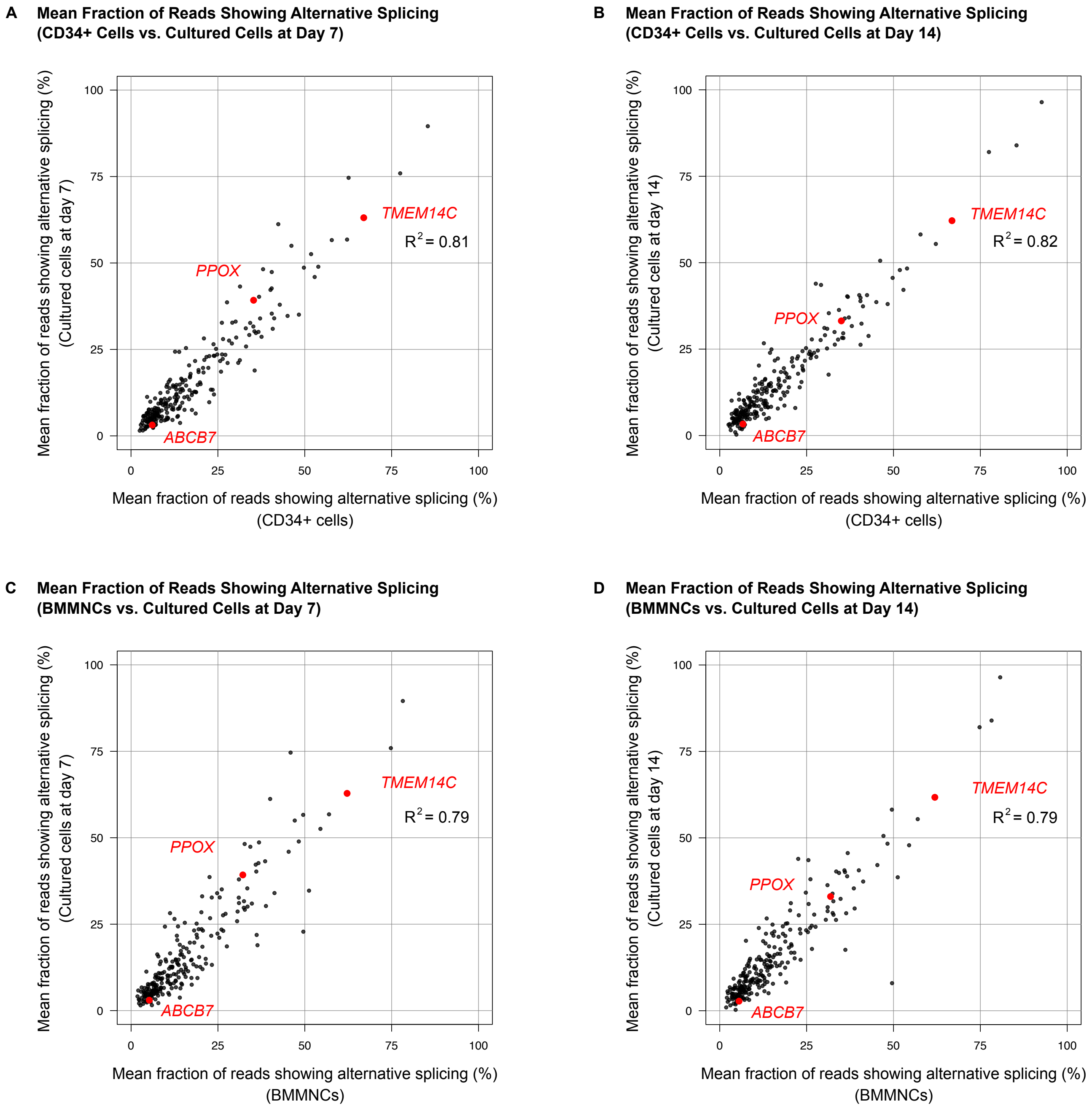

Supplementary Figure 23. Usage of the *SF3B1*-associated abnormal 3′ splice sites in erythroid cells

Scatter plots comparing mean PSI values of *SF3B1*-associated alternative 3′ splice sites between the *SF3B1*-mutated CD34+ cell samples and those from cultured erythroid cells at day 7 (Panel A) and at day 14 (Panel B), and between the *SF3B1*-mutated BMMNCs and those from cultured erythroid cells at day 7 (Panel C) and day 14 (Panel D). Genes related to heme biosynthesis are depicted as red dots.

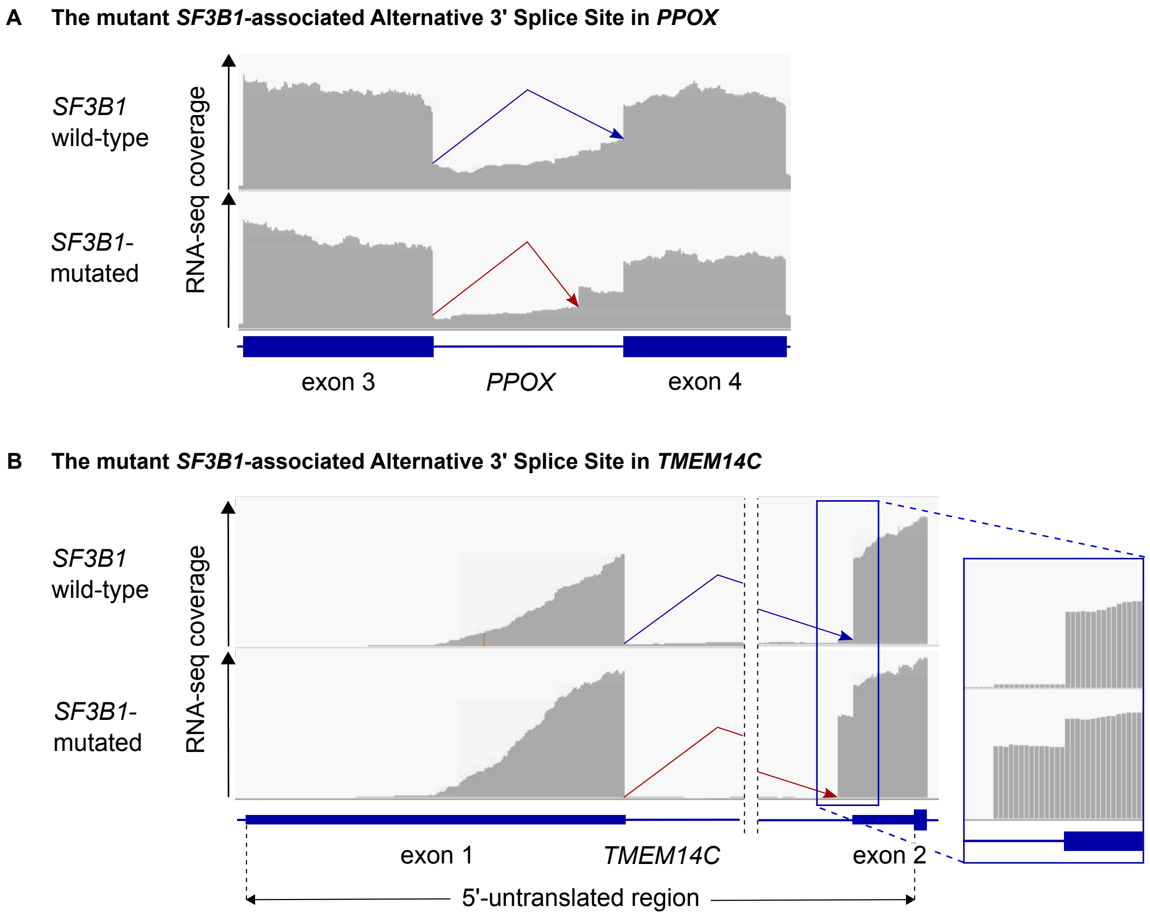

Supplementary Figure 24. Mutant *SF3B1*-associated alternative 3´ splice sites in genes related to heme biosynthesis

Panels A and B show mutant *SF3B1*-associated alternative 3´ splice sites in *PPOX*, and *TMEM14C*, respectively. RNA-seq coverage is shown for samples with mutated and wild-type *SF3B1*.

A Schematics of the Luciferase Reporter Construct

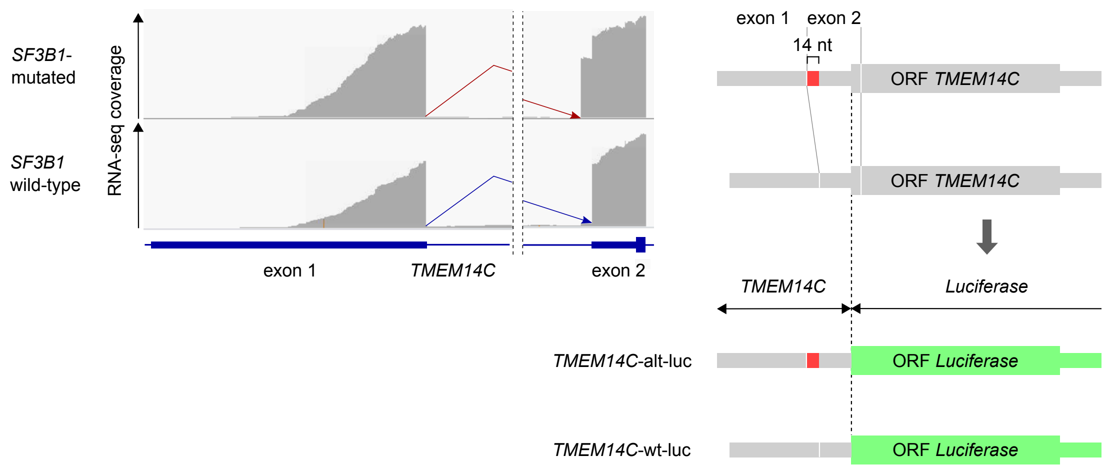

B Relative Luciferase Activity

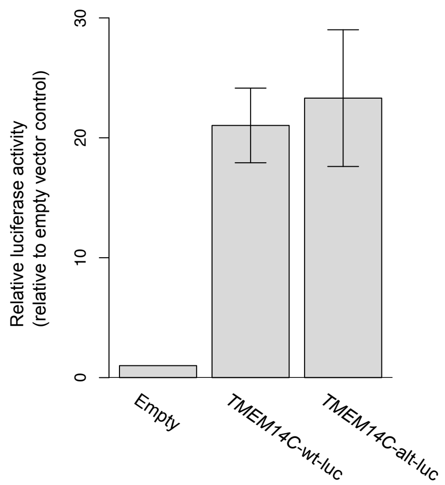

Supplementary Figure 25. Functional significance of the altered 5´-untranslated region of *TMEM14C*

A. Schematics of the luciferase reporter construct. Mutant *SF3B1*-associated usage of the *TMEM14C* alternative 3´ splice site results in insertion of a 14 bp sequence in the 5´-untranslated region (UTR). The 5´-rapid amplification of cDNA end products with and without the mutant *SF3B1*-associated splicing alteration were cloned into pGL3-Basic vector directly upstream of the open reading frame (ORF) of firefly luciferase (denoted as *TMEM14C*-alt-luc and *TMEM14C*-wt-luc, respectively).

B. Firefly luciferase activity normalized against Renilla luciferase activity in HEK293T cells transfected with either empty construct (Empty), pGL3-Basic vector inserted with *TMEM14C* 5´-UTR with or without the mutant *SF3B1*-associated splicing alteration.

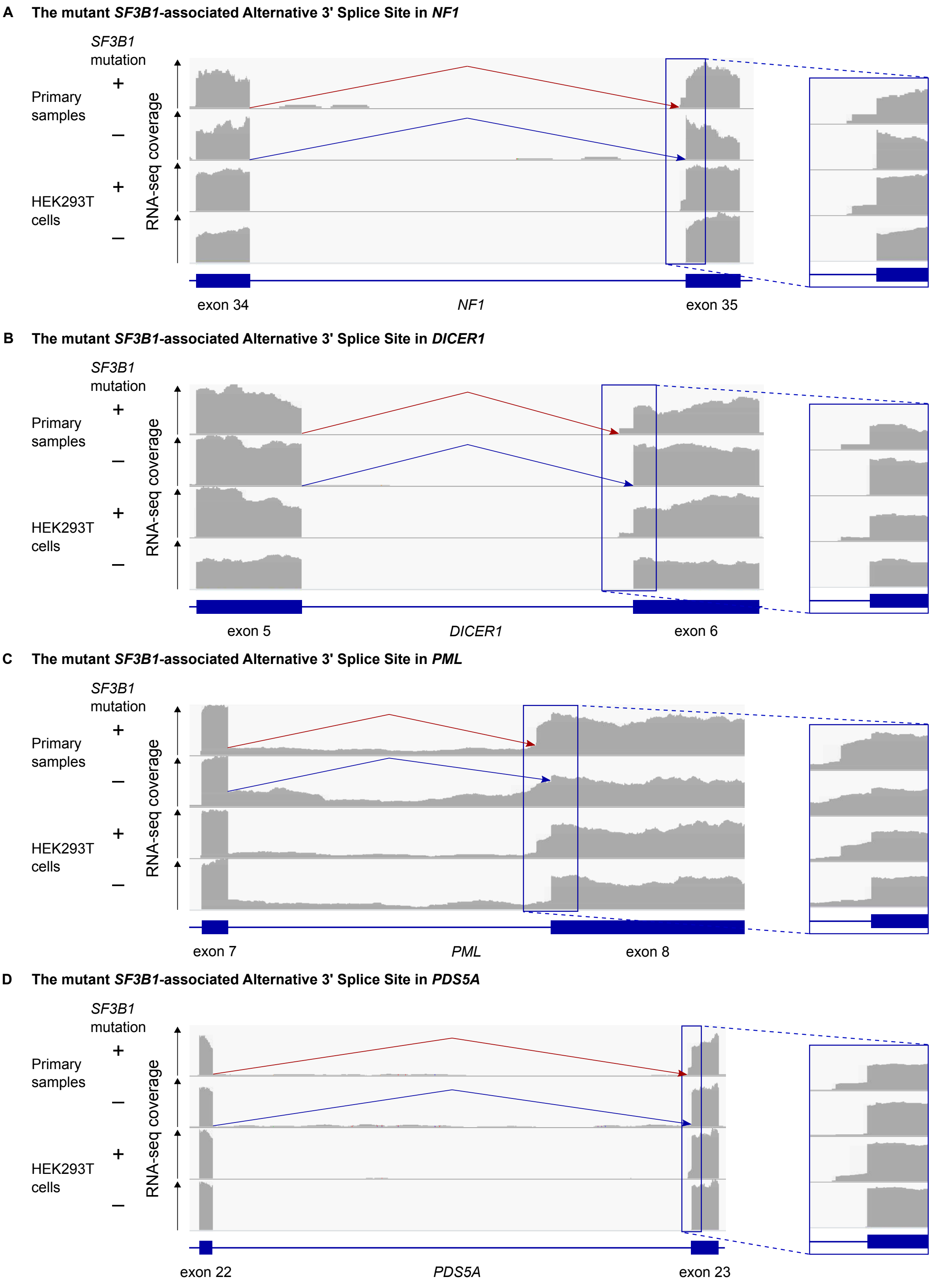
A The mutant SF3B1-associated Alternative 3' Splice Site in NF1
SF3B1 mutation
Primary samples
HEK293T cells
RNA-seq coverage
+
–
exon 34
NF1
exon 35
B The mutant SF3B1-associated Alternative 3' Splice Site in DICER1
exon 5
DICER1
exon 6
C The mutant SF3B1-associated Alternative 3' Splice Site in PML
exon 7
PML
exon 8
D The mutant SF3B1-associated Alternative 3' Splice Site in PDS5A
exon 22
PDS5A
exon 23

Supplementary Figure 26. In vitro validation of mutant *SF3B1*-induced abnormal splicing

Panels A, B, C, and D show mutant *SF3B1*-associated alternative 3´ splice sites in *NF1*, *DICER1*, *PML*, and *PDS5A*, respectively. RNA-seq coverage is shown for primary MDS samples with mutated and wild-type *SF3B1*, as well as for HEK293T cells with and without an *SF3B1*^{K700E} allele introduced by CRISPR/Cas9-mediated gene editing.

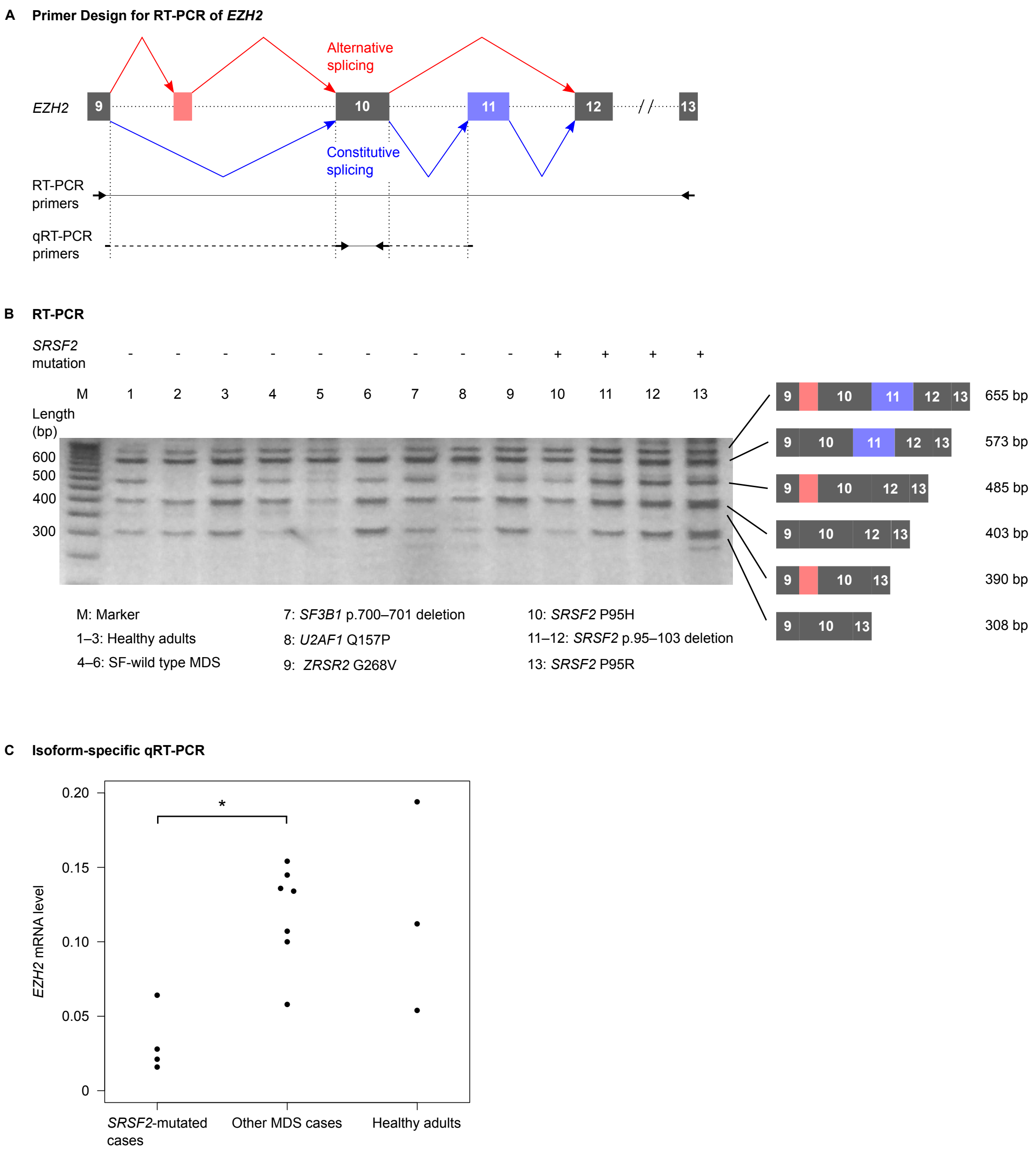

Supplementary Figure 27. Mutant *SRSF2*-associated alternative exon usage in *EZH2*

A. A schematic of mutant *SRSF2*-associated alternative exon usage in *EZH2*. Red and blue arrows indicate alternative and constitutive splicing, respectively. Primer pairs used for RT-PCR and isoform-specific quantitative RT-PCR are indicated as arrows.

B. RT-PCR of the alternatively spliced region of *EZH2* in primary MDS samples. Mutation status of SFs is shown. Alternative isoforms of *EZH2* and lengths of their PCR products are also depicted.

C. Quantitative RT-PCR of the normal transcripts of *EZH2*. Levels of the *EZH2* normal transcripts in bone marrow CD34+ cells are evaluated by isoform-specific quantitative RT-PCR using the primer pair shown in Panel A. *: $P < 0.05$.

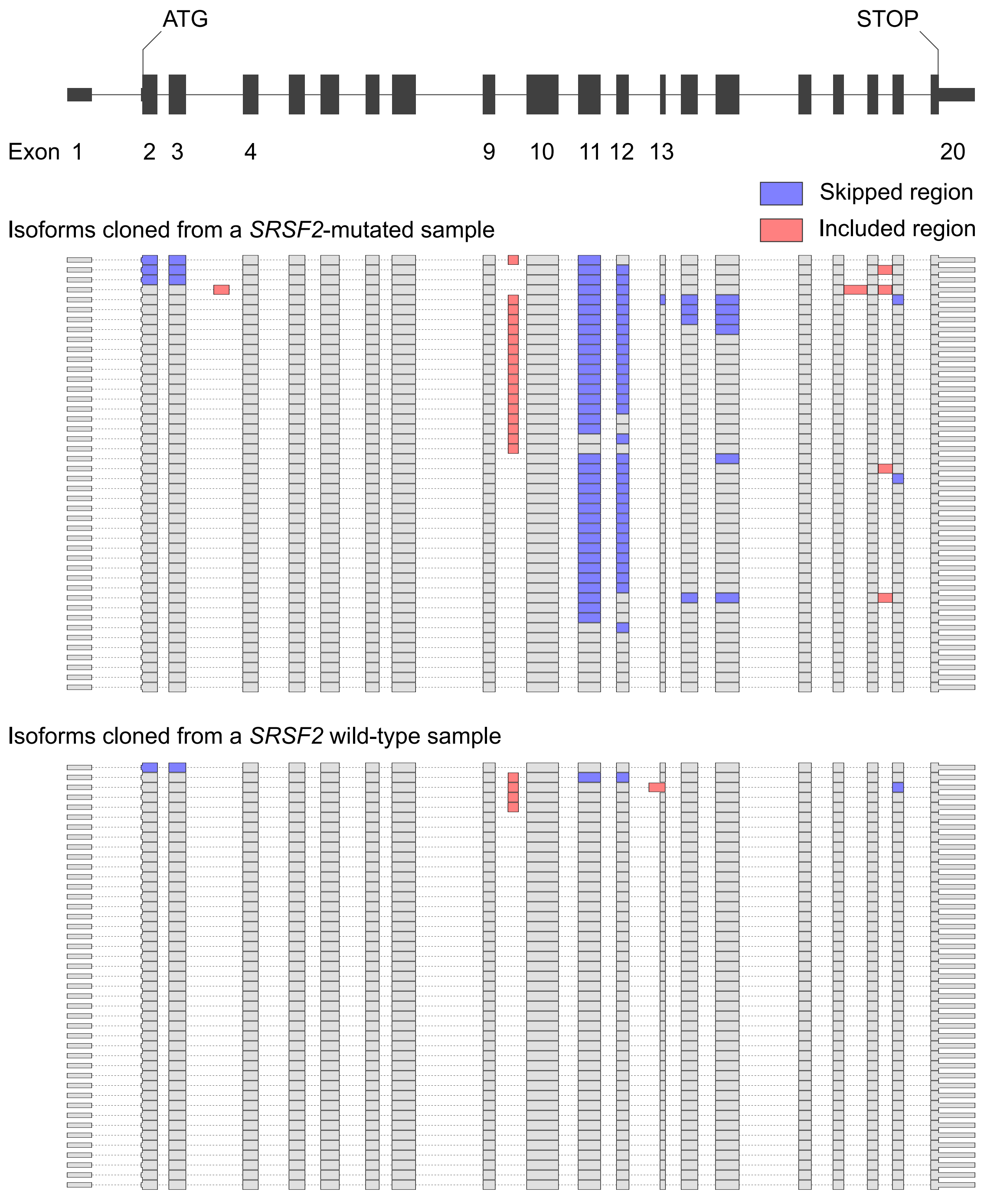

Supplementary Figure 28. Cloning of the entire coding region of *EZH2*

Schematics show the entire coding region of *EZH2* cloned from a *SRSF2*-mutated sample (upper panel, n = 44) and from a *SRSF2* wild-type sample (lower panel, n = 43). The canonical isoform of *EZH2* is shown at the top. Blue and red boxes indicate skipped and included regions, respectively. Mutant *SRSF2*-associated alternative splicing events are inclusion of exon between exons 9 and 10 and skipping of exon 11.

Pathway Analysis (PRC2 targets)

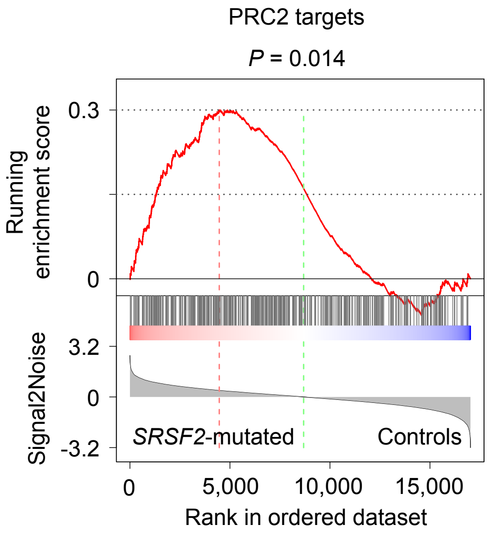

Supplementary Figure 29. Up-regulation of the targets of polycomb repressive complex 2 in *SRSF2*-mutated CD34+ cells

An enrichment plot for known target genes of polycomb repressive complex 2 comparing the *SRSF2*-mutated CD34+ cell samples to those without *SRSF2*, *EZH2*, and *ASXL1* alterations.

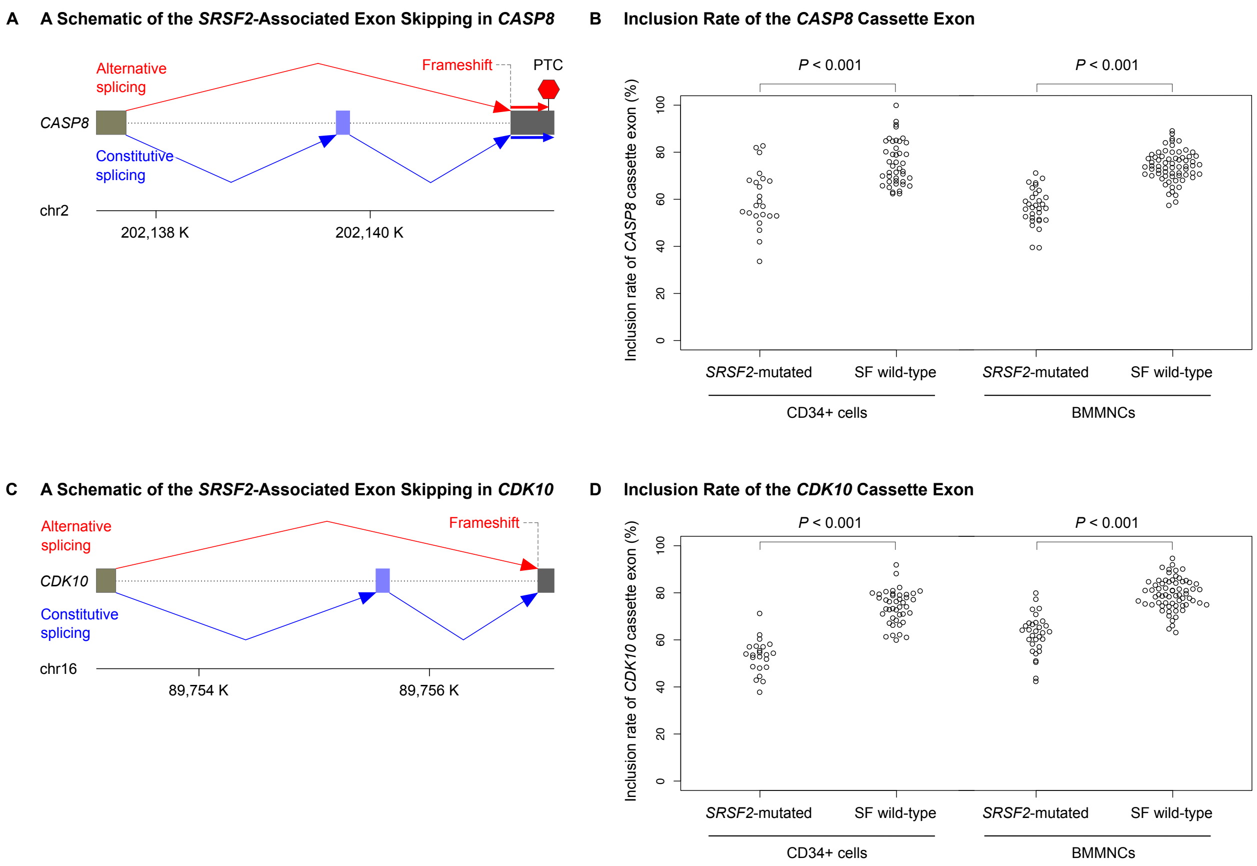

Supplementary Figure 30. Examples of mutant *SRSF2*-associated alternative exon usage

A, C. Schematics of mutant *SRSF2*-associated alternative exon usage in *CASP8* (Panel A), and *CDK10* (Panel C). Gray boxes indicate constitutive exons. Blue boxes denote exons with decreased usage in the *SRSF2*-mutated samples. Red and blue arrows indicate alternative and constitutive splicing, respectively. PTC indicates premature termination codon.

B, D. Dot plots of inclusion rate of mutant *SRSF2*-associated cassette exons in *CASP8* (Panel B), and *CDK10* (Panel D).

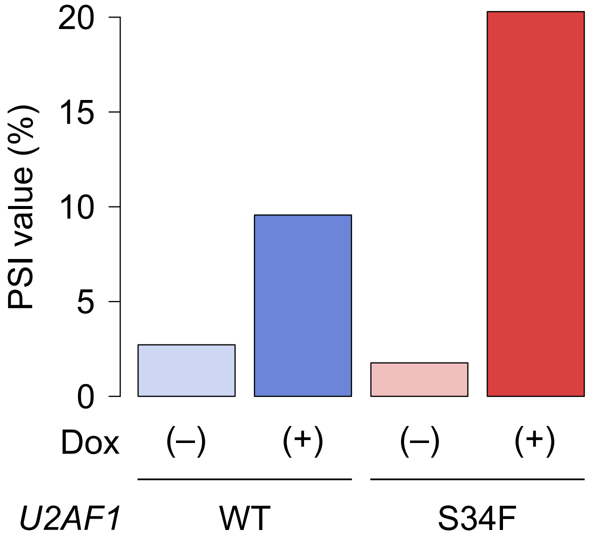

Supplementary Figure 31. Usage of the *EZH2* cryptic exon in HeLa cells transduced with *U2AF1* S34F mutant

A bar plot shows PSI values of the *EZH2* cryptic exon in HeLa cells transduced with doxycycline-inducible *U2AF1* constructs. PSI values were calculated from published RNA sequencing data of cells before and after doxycycline (Dox) induction. HeLa cells had been transduced with wild-type (WT) *U2AF1* or S34F mutant.

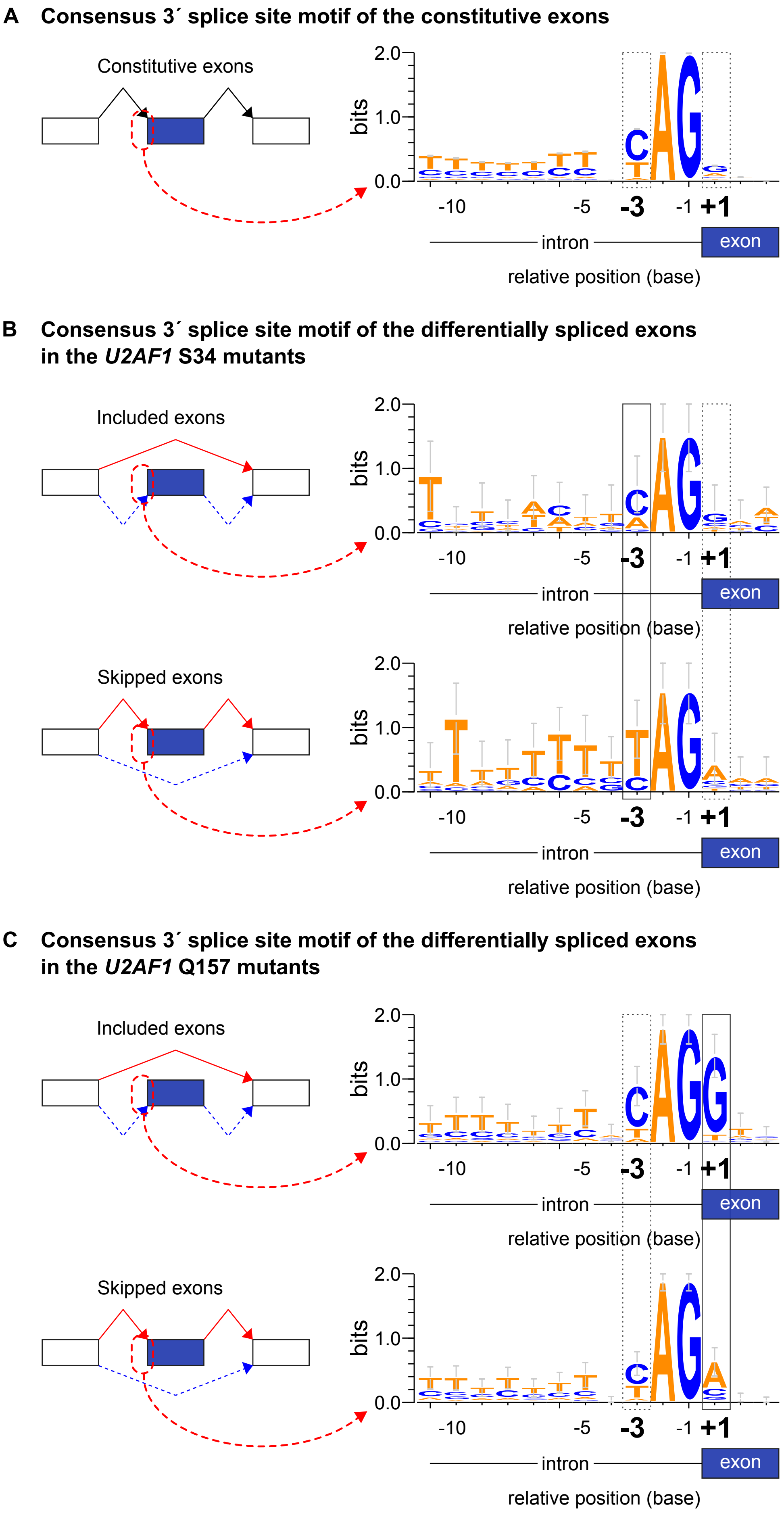
A Consensus 3′ splice site motif of the constitutive exons
Constitutive exons
bits
2.0
1.0
0.0
-10
-5
-3
-1
+1
intron
exon
relative position (base)
B Consensus 3′ splice site motif of the differentially spliced exons in the *U2AF1* S34 mutants
Included exons
Skipped exons
C Consensus 3′ splice site motif of the differentially spliced exons in the *U2AF1* Q157 mutants
Included exons
Skipped exons

Supplementary Figure 32. Mutant *U2AF1*-associated alterations in 3´ splice site consensus sequences

Consensus sequences around 3´ splice sites of constitutively spliced introns (A) and differentially spliced introns in *U2AF1* S34 (B) and Q157 mutants (C). Horizontal axis denotes genomic coordinates defined with respect to the 3´ splice sites. Vertical axis indicates information content in bits. In the panels B and C, more frequently included and skipped exons are drawn separately.

Supplementary Figure 33. Uncropped gel images of RT-PCR gels of differentially spliced introns

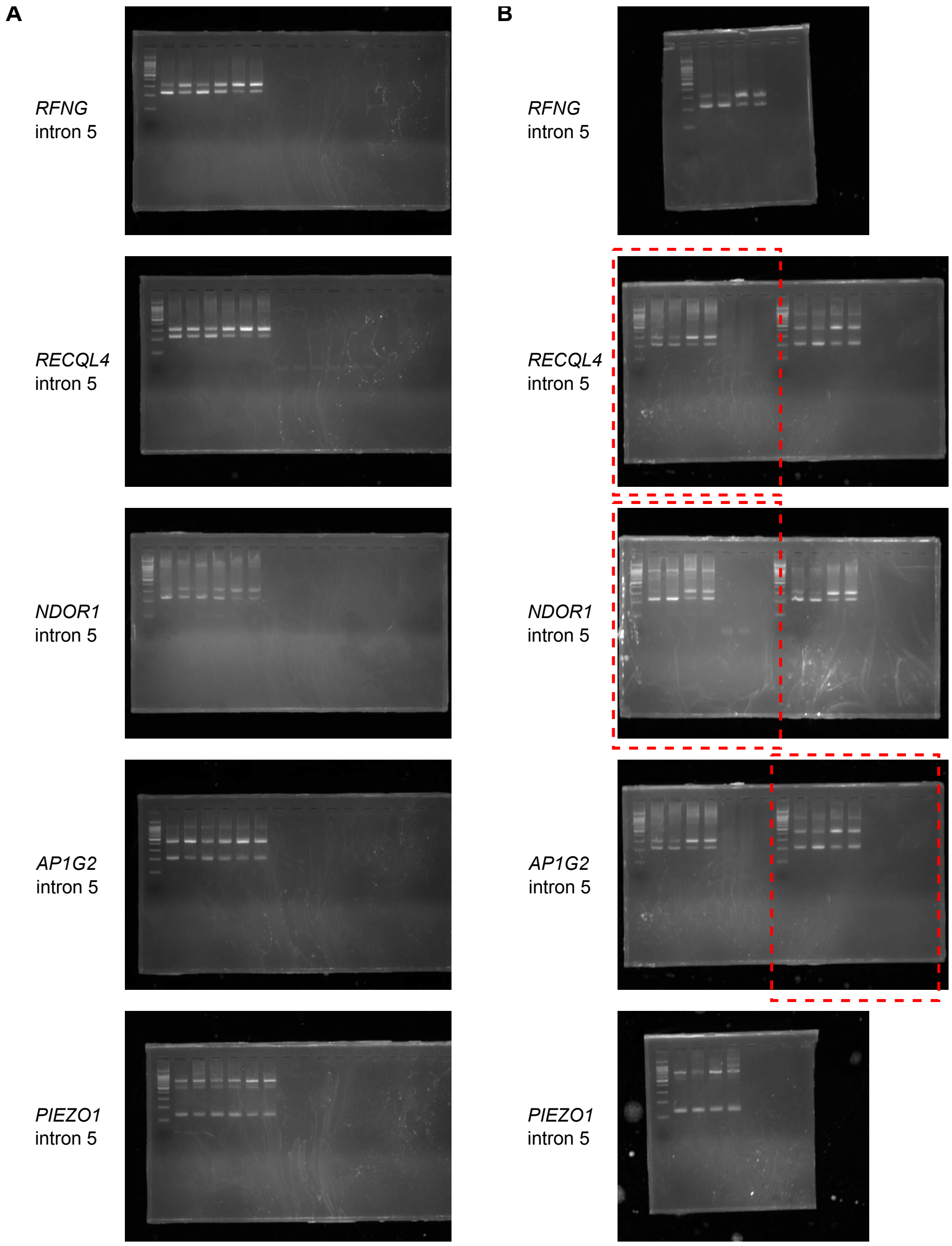

Uncropped gel images of RT-PCR shown in Figs. 6a (panel A) and 6b (panel B). Samples are shown in the original figures. When multiple sample sets were run in the same gel, a relevant part is indicated by a rectangle.

Supplementary Figure 34. Uncropped gel images of RT-PCR gels of the differentially spliced sites of *ABCB7* and *PPOX*

A

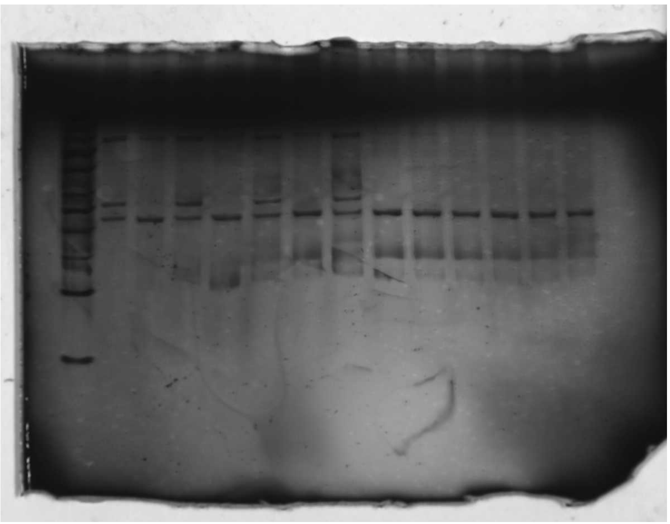

B

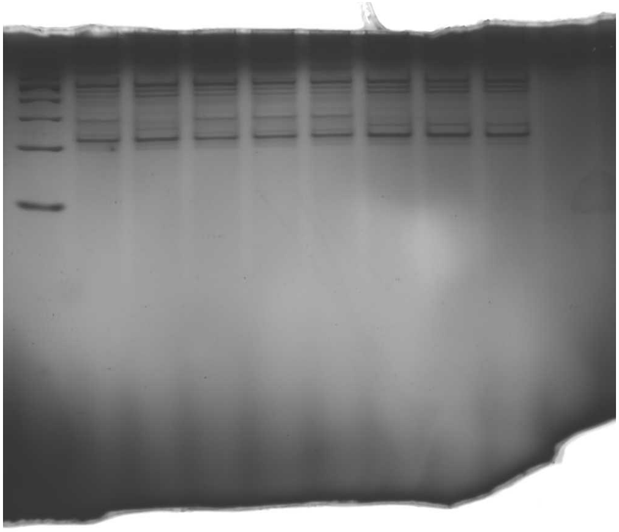

Uncropped gel images of RT-PCR shown in Figs. 7c (panel A) and 7d (panel B). Samples are shown in the original figures.

Supplementary Figure 35. Uncropped images of immunoblots of CRISPR cell lines

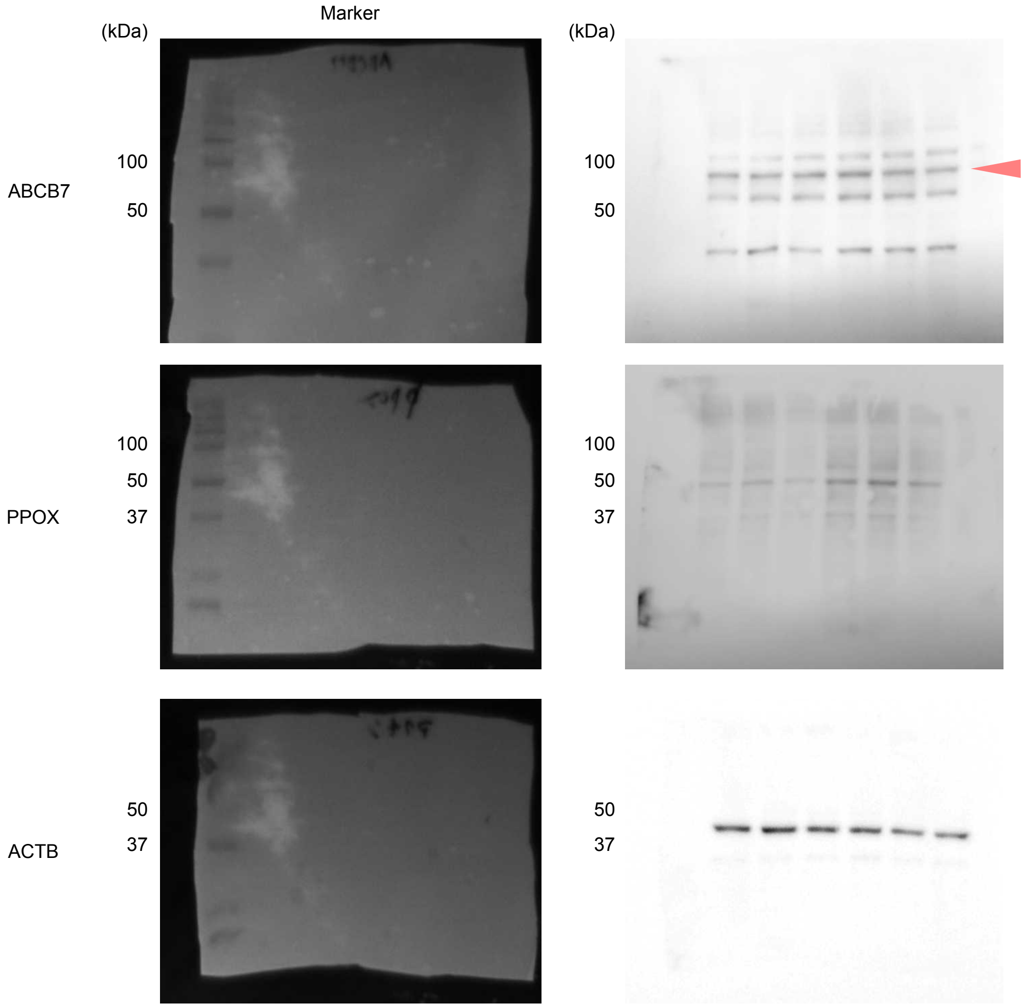

Uncropped images of immunoblots shown in Fig. 7e. Samples are shown in the original figures.

Non-luminescent markers were scanned under direct light, and were shown in the left half.

2. Supplementary Tables

Supplementary Table 1. Patient characteristics

Number of cases	214
Age (median, range)	67 (30–91)
Sex (male/female)	132/82
Diagnosis	
MDS	152
MDS-SLD	11
MDS-RS-SLD	34
MDS-MLD	39
MDS-RS-MLD	17
MDS with isolated del(5q)	4
MDS-EB-1	22
MDS-EB-2	25
MDS/MPN	44
CMML-1	32
CMML-2	1
MDS/MPN-RS-T	8
MDS/MPN-U	3
Acute myeloid leukemia with myelodysplasia-related changes	18
Hemoglobin (g/dL) (median, range)	9.9 (6.3–15.5)
WBC count (x 10^9/L) (median, range)	4.6 (0.8–61.6)
Absolute neutrophil count (x 10^9/L) (median, range)	2.1 (0.2–32.0)
Platelet count (x 10^9/L) (median, range)	146 (13–939)
Bone marrow blasts (%) (median, range)	3 (0–90)
Bone marrow ring sideroblasts (%) (median, range)	5 (0–95)

MDS indicates Myelodysplastic syndromes; MDS-SLD, MDS with single lineage dysplasia; MDS-RS-SLD, MDS with ring sideroblasts with single lineage dysplasia; MDS-MLD, MDS with multilineage dysplasia; MDS-RS-MLD, MDS with ring sideroblasts with multilineage dysplasia; MDS-EB, MDS with excess of blasts; MDS/MPN, Myelodysplastic/myeloproliferative neoplasm; CMML, chronic myelomonocytic leukemia; MDS/MPN-RS-T, myelodysplastic/myeloproliferative

neoplasm with ring sideroblasts and thrombocytosis; MDS/MPN-U, myelodysplastic/myeloproliferative neoplasm, unclassifiable; WBC, white blood cell.

Supplementary Table 2. Gene list for targeted deep sequencing

ACSM2A	*EZH2*	*PIGA*
ALDH1B1	*FANCA*	*PIGT*
ARID2	*FANCM*	*PPM1D*
ASXL1	*FLT3*	*PRPF8*
ASXL2	*GATA2*	*PTPN11*
ATM	*GIGYF1*	*PXDNL*
ATRX	*GNAS*	*RAD21*
BCOR	*GNB1*	*RIT1*
BCORL1	*IDH1*	*RUNX1*
BOD1L1	*IDH2*	*SETBP1*
BRCC3	*IRF1*	*SETD2*
CALR	*JAK2*	*SF1*
CBL	*JARID2*	*SF3A1*
CDH23	*KDM6A*	*SF3B1*
CDKN2A	*KIT*	*SH2B3*
CEBPA	*KMT2D*	*SMC1A*
CHEK2	*KRAS*	*SMC3*
CLCN6	*LTN1*	*SRSF2*
CREBBP	*LUC7L2*	*STAG1*
CSF3R	*MPL*	*STAG2*
CTCF	*MRE11A*	*STAT3*
CUX1	*NEURL*	*TERT*
DCLRE1C	*NF1*	*TET2*
DDX41	*NFE2*	*TP53*
DNMT3A	*NPM1*	*U2AF1*
DST	*NRAS*	*U2AF2*
DYNC2H1	*NRIP1*	*USP9X*
EP300	*NXF1*	*WT1*
ETNK1	*PDS5B*	*ZRSR2*

Supplementary Table 3. Number of SF-mutated patients with or without mutations in epigenetic regulators

SF	Epigenetic regulator	Bone marrow CD34+ cells		BMMNCs	
		Double mutant	SF single mutant	Double mutant	SF single mutant
SF3B1	*TET2*	14	18	15	39
	DNMT3A	2	30	5	49
	IDH1/*IDH2*	0	32	0	54
	ASXL1	6	26	9	45
	EZH2	3	29	2	52
SRSF2	*TET2*	13	10	13	19
	DNMT3A	0	23	0	32
	IDH1/*IDH2*	4	19	6	26
	ASXL1	7	16	11	21
	EZH2	0	23	0	32

Supplementary Table 4. Off-targets and primer sequences

Locus	Forward primer sequence	Reverse primer sequence
chr11:27679873	CCCATGGGATTGCACTTGG	CTCCCTACAGTTCCACCAGG
chr8:74970580	TGCCTGGGAGACTCCTATGA	AGATTGCGCCACTGCCTT
chr1:226589938	AGTCCAGGAGGTGTTGCTG	AGGTCAAGGTCTAGTGGGTCT
chr13:111589339	TGGAGTACACCAAGAGCGG	TTGGATAGGCGCATCTGGC
chr21:17967356	ATGCTAAAGCCCAGCCTGG	GCACTAGGTGGCCCTCACTA
chr4:166649304	AGGAGAATGCCCTTCCATAGG	AGCTGGACCATGCATTCTTCA
chr21:22930086	GCGTTATGAATCTGGGTGCCAC	ACAAGAGGTCCTTCAGGTAGCC
chr1:172797724	ACATTTTCAGAGCCATGCTGC	AGGTGAATGCACTTCGGCA
chr6:77602520	TCTGCATACTCTCCATGTGTGT	TGACCTTAGGCAAGCTGCT
chr22:25515699	TCATAGGAGCAGGGAGACACA	ATCAGAATCCCCCTCCCTGG

Supplementary Table 5. Primer sequences for RT-PCR, PCR cloning, qRT-PCR, and 5′-RACE

Usage	Gene	Forward primer sequence	Reverse primer sequence
RT-PCR	*ABCB7*	AATGAACAAAGCAGATAATGATGCAGG	TCCCTGACTGGCGAGCACCATTA
RT-PCR	*PPOX*	GGCCCTAATGGTGCTATCTTTG	CTTCTGAATCCAAGCCAAGCTC
RT-PCR	*EZH2*	AACCTTGTGGACCACAGTGTT	TCAGCTGTATCTTTCTGCAGTG
RT-PCR	*RFNG*	GACCACGGTCAAGTTCTGGTTTG	CCTCTGCAGGTTCTCCAGGTG
RT-PCR	*RECQL4*	CCTGCCACCGTGTCTCAAGG	GAAGTTGTGGGACCACTGGGAG
RT-PCR	*NDOR1*	CTTCGAACTCCTGGCCTGTCTAT	CGAGGAGGCGATGGAGAAGG
RT-PCR	*AP1G2*	CGAAGAGGAAGTGCTGGCATTG	CCGGAAGAGTGTGTCATACTCCA
RT-PCR	*PIEZO1*	TTGTCAACCCCCAGGAGTATTCCAG	CTTTCCGCACCCCAAACCAGTTG
PCR cloning	*EZH2*	CTAATACGACTCACTGGACTCAGAAGGCAGTGGAG	GATTACGCCAAGCTTCAGCTGTTTCAGAGGAGGGG
qRT-PCR	*GAS5*	CTTGCCTGGACCAGCTTAAT	CAAGCCGACTCTCCATACCT
qRT-PCR	*UHG*	GGCCAGCACCTTCTCTCTAA	TCCTCCAAGACAGATTCCATTT
qRT-PCR	*EZH2*	CAGCATTTGGAGGGAGCAAAG	ACCGAGAATTTGCTTCAGAGGA
qRT-PCR	*ACTB*	CATTGGCAATGAGCGGTTC	CGTGGATGCCACAGGACT
5′-RACE	*TMEM14C*	Primer 1: GATTACGCCAAGCTTTGCCAGCCAAGGTACCAGATGTAGCTAGGA	
		Primer 2: GATTACGCCAAGCTTGCTCTTGTGTTACATACTCGCCACCTCTGT	

Supplementary Table 6. Immunoblots primary antibodies, dilutions used in experiments, company, and catalogue information.

Epitope	Company	Catalog number	Clone name	Host	Dilution
Actin	Santa Cruz Biotechnology	sc-1616	Polyclonal	Goat	1:5000
ABCB7	GeneTex	GTX114916	Polyclonal	Rabbit	1:5000
PPOX	Sigma-Aldrich	WH0005498M1	2F10	Mouse	1:1000

3. Supplementary Methods

Variant filtering criteria

Variants were filtered as in the previous paper and oncogenic variants were identified[1]. Putative variants were first annotated using the following database: 1000 Genomes Project released in April 2012, ESP6500, CG69, dbSNP version 137, Catalogue of Somatic Mutations in Cancer (COSMIC) version 67, ClinVar released at November 5th, 2013, and our in-house SNP database. Variants that fulfilled the following criteria were removed:

1. variants that do not have an effect on an amino acid sequence except for *TERT* promoter mutation;
2. variants of which an allele frequency < 0.05;
3. variants with a sequencing depth < 50;
4. variants that were registered to our in-house SNP database;
5. variants with a population frequency > 0.0014 (based on incidence of myeloid malignancies in the population and of a known driver event *JAK2* V617F in public databases) unless they were registered to confirmed somatic mutation in COSMIC;
6. variants within regions prone to sequencing errors, including regions of high depth and repeat elements;
7. highly recurrent calls with narrow allele frequency distribution in both tumor and control samples. This can either be an allele frequency of <10% indicative of artifacts or that of ~50% indicative of polymorphisms.

Target genes of the polycomb repressive complex 2

A gene set of the polycomb repressive complex 2 targets was defined as follows according to the previous paper[2]: *ABCC8, ABTB2, ADAMTS15, ADAMTS18, ADARB2, ADCY4, ADCY8, ADCYAP1, ADRA1A, ADRA2A, ADRB1, ADRB3, ALOX15, ALX3, ALX4, ANKRD19, ANKRD20A1, ANKRD20B, ANKRD27, AQP5, ARHGAP20, ARL9, ASCL1, ASCL2, ASTN1, ASTN2, ZFHX3, ATF3, ATOH1, ATOH8, NKX3-2, BARHL1, BARHL2, BARX1, BARX2, BCL2, BHLHE41, BHLHE23, BHLHE22, BMP8A, BNC1, BTG2, MKX, TMEM59L, FAM89A, ILDR2, FAM163A, LRRC71, LAMP5, EVA1C, CNRIP1, MAATS1, CA10, CACNA1B, CACNA1D, CACNA1E, CACNA1G, CALCA, CAMK2N1, CASZ1, CBLN1, CBLN4, CBR3, CBX8, CD34, CD8A, CDH23, CDH7, CDK5R2, CDKN2C, CDX2, ADAP2, CGB7, CGB8, TPPP3, CH25H, CHODL, CHRD, CHRDL2, CHST8, VSX2, CIDEA, CITED1, CMTM2, CLCN5, CLEC14A,*

CLSTN2, CNNM1, CNTFR, COL24A1, COL25A1, COL27A1, COL2A1, COL4A5, COL4A6, COL9A2, COLEC12, COMP, CORO6, CRHR1, CRLF1, CRTAC1, CRYBA2, CSMD1, CSMD3, CTNND2, GJA9, CXCL14, CXCL16, CYP24A1, CYP26A1, CYP26B1, CYP26C1, CYP27B1, DACH1, DACH2, DCLK2, DCC, DCHS2, DDAH1, DGKG, DGKI, DHH, DIO3, PARM1, DKK1, DKK2, DLL4, DLX1, DLX2, DLX3, DLX4, DMRT1, DMRT2, DMRT3, DOK6, DPF3, DPY19L2, DRD5, DSC3, DSCAML1, DUOX1, DUOX2, DUSP4, ECEL1, EFNA1, EFNA3, EGFL6, EGR3, EGR4, ELMOD1, EN1, EN2, EOMES, EPAS1, EPB41L4A, EPHA5, EPHB1, EPHB3, ERBB4, ESAM, ESPN, ESX1, F2R, FAM19A4, FAM43B, FAM5B, FAM5C, RIMKLA, FAM84A, FBN2, FBP1, FBXL8, FBXO3, FEV, FEZ1, FGF20, FGF3, FGF5, FGF9, FIGLA, FLI1, FLJ11235, FLJ13236, TET2, FLJ32063, CCDC140, FLJ33790, SLFN11, FLJ35409, ANKRD18A, DPY19L2P2, FBLN7, C8orf47, FLJ44815, FLJ45455, FLJ45983, FLJ46347, FLRT2, FOXA2, FOXB1, FOXD2, FOXD3, FOXD4L4, FOXD4L1, FOXD4L2, FOXD4L3, FOXE1, FOXF1, FOXG1, FOXJ1, FOXL1, FOXL2, FRMD3, FUT4, FZD10, FZD2, GABRA2, GABRA4, GAD2, B4GALNT1, B4GALNT2, GALNT18, GALR2, GATA2, GATA3, GATA4, GATA6, GBX2, GDF6, GDF7, GDNF, GHR, GHSR, GIMAP5, GJB2, GLT25D2, GNA14, GPC5, GPM6B, PRLHR, GPR101, GPR12, FFAR4, GPR88, GRIA2, GRID1, GRIK1, GRIK3, GRIN3A, GRM7, GSC, GSC2, GSX1, GSX2, GUCY1A3, GUCY2D, HAND2, HBA1, HBA2, HES2, HES7, HEY1, HHAT, HHEX, HHIP, HLX, MNX1, HMX2, HMX3, HOXB1, HOXB13, HOXB2, HOXB3, HOXB6, HOXB7, HOXB8, HOXC11, HOXC12, HOXC4, HOXC5, HOXC6, HOXC8, HOXD1, HOXD12, HOXD13, HOXD3, HOXD4, HOXD8, HOXD9, HPCAL4, HPSE2, HRK, HS3ST3B1, HS6ST1P, HS6ST3, HSF4, HSPA6, HTR1A, HTR2C, HTR7, ICAM5, IGF2AS, IGSF21, IL1RAPL2, IL7, INA, INSM2, INSRR, PDX1, IRX3, IRX4, IRX5, ISL1, ISL2, ITGA4, ITPKA, JUN, KAZALD1, KCNA1, KCNA3, KCNAB1, KCNC2, KCNC4, KCND3, KCNH1, KCNH3, KCNK12, KCNK13, KCNK2, KCNK4, KCNMA1, KCNQ3, KCNV1, VASH1, KIAA1199, KIAA1324, RIMBP3, KIRREL3, KL, KLF4, KY, LBX1, LGALS3, LGR5, LHX2, LHX4, LHX5, LHX6, LHX8, LMX1B, TMEM132E, C1orf194, LAYN, C1orf213, LOC150221, LOC153684, NBPF11, PABPC1L2A, RPRML, C17orf82, ANXA2R, LOC400120, DUOXA2, LOC440804, LOC441413, ANKRD20A3, LOC441426, ANKRD20A2, ANKRD18B, NDUFA4L2, TMEM88, LPHN3, LPL, LRCH2, LRFN5, LRP2, LRRTM1, LTBP2, LTK, LYSMD2, MAB21L1, MAB21L2, MAFB, MAL, MAPK4, MAPT, MCOLN3, MESP1, METRNL, AGPAT9, FAM81A, MGC26718, RSPO2, MGC39545, MLLT3, MSC, MSX1, MT1A, MT1B, MT1H, MT1M, MT1DP, MYF6, MYO5B, MYOD1, NAGS, NAV2, NCAM1, NEFM, NEFL, NELL1, NEUROD1, NEUROD2, NEUROG1, NEUROG2, NEUROG3, NFIX, DUOXA1, NKX2-2, NKX2-3, NKX2-8, NKX3-1, NKX6-1, NKX6-2, C2CD4A, NOL4, NPAS1, NPNT, NPR3, NPTX1, NPY1R, NR2F2,

NR4A3, NRG1, NRG2, OAF, NT5C1A, NTN1, NTNG2, NTRK1, NTRK2, NPAS4, OCA2, OLFML2B, OLIG2, ONECUT1, ONECUT2, OPRD1, MGARP, OSR1, OTOP1, OTOP2, OTOP3, OTP, OTX1, OTX2, OXCT2, PAPPA, PAX1, PAX2, PAX3, PAX6, PAX7, PAX8, PAX9, PCDH17, PCDH8, PDE4DIP, PDGFRA, FRMPD4, PDZD2, PENK, PGM5, PGR, PHOX2A, PHOX2B, PIP5K1B, PIR, PITX1, PITX2, PITX3, PKNOX2, PKP1, PLEC, PLXNA2, PMP22, PODN, POLE, POU3F1, POU3F4, POU4F1, POU4F2, POU4F3, PPM1E, PRAC, PRDM12, RP11-35N6.1, PRKCE, PRKG1, PROK2, PTF1A, PTGDR, PTGER2, PTGER3, PTGER4, PTGFR, PTHLH, PTPRT, PTPRU, PXMP2, PYY, RAB6C, SHC4, RASGRF1, RASSF5, RAX, RBP4, REPS2, RGCC, RGS10, RGS20, RGS9BP, RIPK3, LONRF3, RNF128, ROBO3, RPS6KA6, RASL10A, RSPO1, RTN4RL2, RYR3, SCD5, SCN4B, SCNN1G, SCTR, SEMA6D, SFRP1, SFRP5, SGPP2, SHH, SHOX, SHOX2, SIDT1, SIM2, SIX1, SIX2, SIX3, SIX6, SLC10A4, SLC1A2, SLC1A4, SLC24A4, SLC26A4, SLC27A2, SLC30A2, SLC30A3, SLC30A4, SLC32A1, SLC35F3, SLC6A1, SLC6A3, SLC6A5, SLC9A2, SLC9A3, SLCO2A1, SLCO5A1, SLIT1, SLIT2, SLITRK1, SLITRK3, PIGZ, BATF3, SORCS1, SORCS3, SOX14, SOX17, SOX7, SPAG6, SPOCK3, SPON1, SRD5A2, SSTR1, SSTR2, ST8SIA2, STK32B, STMN2, STXBP6, SUSD4, SV2B, SYT12, TAL1, TBR1, TBX1, TBX2, TBX21, TBX3, TBX5, TCEA3, HNF1B, TFAP2E, THBD, PTH2, NKX2-1, TLL1, TLX1, TLX2, TMEFF2, TMEM27, TMEM30B, TMOD2, CD70, TP73, TRADD, TRH, TRIM36, TRIM67, TRIM9, TRPC5, TSLP, TTYH1, UCN, UCP1, UNC5C, FAM150A, USH1G, VAX1, VAX2, VDR, VSX1, WRAP73, WT1, WNT1, WNT10A, WNT10B, WNT11, WNT16, WNT2, WNT3A, WNT6, WNT7A, WT1, ZADH2, ZBTB16, ZCCHC16, ZEB2, ZFYVE28, ZIC1, ZIC4, ZMYND15, FEZF2, ZNF436, ZNF503, and *IKZF3*.

4. Supplementary References

1. Papaemmanuil E*, et al.* Clinical and biological implications of driver mutations in myelodysplastic syndromes. *Blood* **122**, 3616-3627; quiz 3699 (2013).

2. Ben-Porath I*, et al.* An embryonic stem cell-like gene expression signature in poorly differentiated aggressive human tumors. *Nat Genet* **40**, 499-507 (2008).
